# An Experimental and Computational Investigation of the Kinetics of Branched Butyl Radicals 2‑Methylpropan-1-yl and 2‑Methylpropan-2-yl with Molecular Oxygen

**DOI:** 10.1021/acs.jpca.6c05216

**Published:** 2026-10-06

**Authors:** Timo T. Pekkanen, Raimo S. Timonen, György Lendvay, William H. Green, Arkke J. Eskola

**Affiliations:** † Department of Chemistry, 3835 University of Helsinki, P.O. Box 55 (A.I. Virtasen aukio 1), 00014 Helsinki, Finland; ‡ Department of Chemical Engineering, Massachusetts Institute of Technology, Cambridge, Massachusetts 02139, United States; § 280964 Institute of Materials and Environmental Chemistry, Research Centre for Natural Sciences, Magyar Tudósok krt. 2., Budapest H-1117, Hungary

## Abstract

We have measured the rate coefficient for the reaction between the 2-methylpropan-1-yl radical and molecular oxygen as a function of temperature and pressure in both He and N_2_ bath gases using photoionization mass spectrometry. Similar measurements were performed for the 2-methylpropan-2-yl radical. It was found that 2-methylpropan-1-yl reacts much slower with O_2_ than 2-methylpropan-2-yl both in the falloff region and near the high-pressure limit. We complemented the experimental work by constructing master-equation models for both reaction systems to simulate their kinetics under conditions not covered by experiments. By optimizing the value of few key parameters against measured data, the models were able to reproduce the observed temperature and pressure dependencies of both rate coefficients. To facilitate the use of present results in modeling, PLOG and Chebyshev rate-coefficient parametrizations are provided. Although 2-methylpropan-1-yl reacts much slower with O_2_ than 2-methylpropan-2-yl, the former reaction generates more products that lead to chain-propagation and -branching than the latter, which almost exclusively forms 2-methylpropene and hydroperoxyl and is thus chain-terminating.

## Graphical abstract

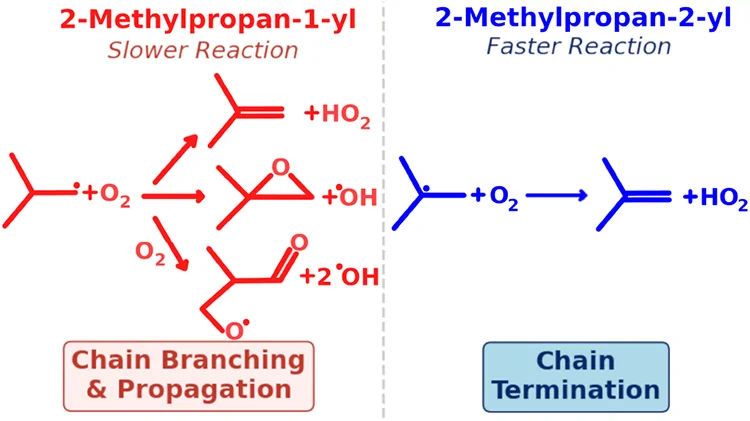

## Introduction

Accurately describing the combustive oxidation of 2-methylpropan-1-yl (*i*-C_4_H_9_^•^, *i*-butyl) and 2-methylpropan-2-yl (*t*-C_4_H_9_^•^, *t*-butyl) is crucial not just because these radicals are directly formed by the decomposition of larger hydrocarbon fuels such as 2,2,4-trimethylpentane (iso-octane), but also because they serve as useful prototypes to model and explain how larger hydrocarbon-fuel radicals oxidize. For low-temperature (*T* < 900 K) combustion (LTC), having a comprehensive understanding of reaction networks and the magnitudes of rate coefficients is particularly important since autoignition chemistry is quite sensitive to the underlying mechanistic details.^1^ C4-hydrocarbons essentially contain all the alkane-oxidation reaction channels present in larger hydrocarbons, and their relatively small size enables researchers to keep cost and complexity under control both in experimental and computational research. C4-radicals can be selectively produced and detected in experimental work, and their small size permits computational investigators to use expensive-but-accurate quantum-chemistry methods.

An interesting feature of the two reactions studied in this work,

R1 $$i\text{−}C_{4}H_{9}^{•}+O_{2}\to \mathrm{Products}\;\mathrm{and}$$

R2 $$t\text{−}C_{4}H_{9}^{•}+O_{2}\to \mathrm{Products}$$

is how differently they contribute to autoignition chemistry in LTC. R2 is known to be chain-terminating in LTC as it almost exclusively forms 2-methylpropene and hydroperoxyl by concerted elimination (conjugate-alkene channel); Atri et al.^2^ measured the yield of this channel to be about 99% at 753 K. As shown in Figure 1b, energetics clearly favor this product channel. The situation is different for R1, in which the barriers for intramolecular hydrogen-abstraction reactions are lower than the one for concerted elimination (see Figure 1a). In a computational study, Xing et al.^3^ found the yields of the chain-propagating 2,2-dimethyloxirane + hydroxyl (P2) and chain-terminating methylpropene + hydroperoxyl (P1) channels to be comparable. Based on the reaction-energy profiles shown in Figure 1, QOOH stabilization is expected to always be negligible for R2, but for R1, enough QOOH can be formed at high *p* and/or [O_2_] to introduce second-O_2_-addition chemistry that leads to chain-branching.

**Figure 1:**
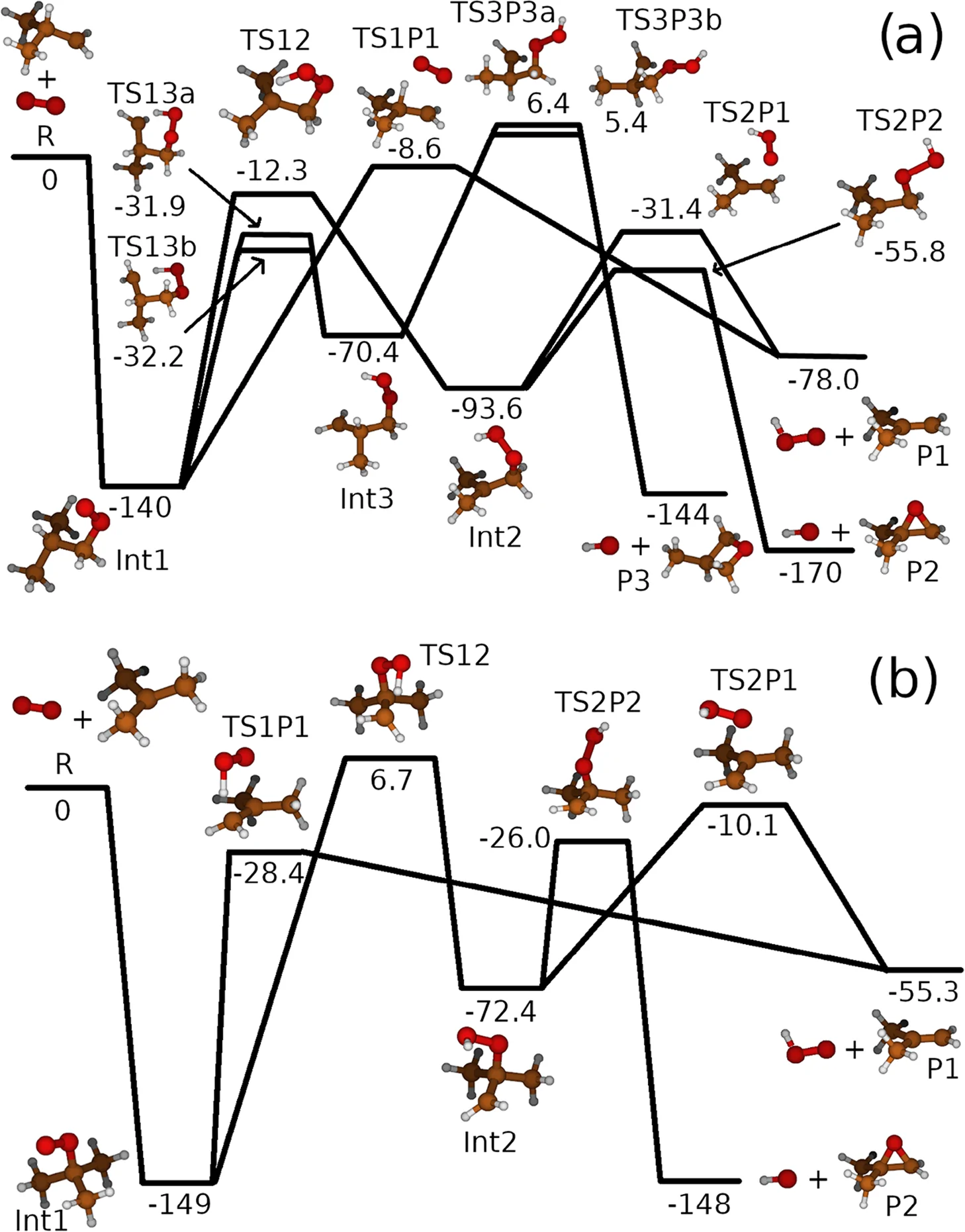
Zero-kelvin reaction-enthalpy profiles for the (a) *i*-C_4_H_9_^•^ + O_2_ and (b) *t*-C_4_H_9_^•^ + O_2_ reaction systems. All energies are in kJ mol^–1^ and were obtained at the ROHF-CCSD­(T)/CBS//MN15/Def2TZVP level of theory + various additive corrections. See text for details.

Experimental data for R1 are scarce; we found only one direct measurement of its rate coefficient.^4^ Wu and Bayes performed room-temperature measurements in 2.3 and 3.5 Torr of He or N_2_. The radical was produced by flash photolyzing (FP) 3-methylbutyl nitrite and detected with photoionization mass spectrometry (PIMS) under pseudo-first-order conditions, and they obtained *k*_1_ = (2.9 ± 0.7) × 10^–12^ cm^3^ s^–1^, independent of pressure and bath gas. Given that pressure dependence has been observed for other C4 hydrocarbon radicals at 1–10 Torr,^5,6^ it does not seem plausible that R1 should be pressure independent at such low pressures. More data are available for R2. The bulk of the experimental data come from a previous study by our group.^5^ The Bayes group also performed room-temperature FP-PIMS measurements for R2,^7^ and again, obtained a pressure independent value of *k*_2_ = (23.4 ± 3.9) × 10^–12^ cm^3^ s^–1^. Dilger et al.^8^ determined the rate coefficient of R2 at 241–462 K and 1.5 bar (N_2_) using the muon-spin-relaxation technique in longitudinal magnetic fields and report a room-temperature value of *k*_2_ = (17.9 ± 5.0) × 10^–12^ cm^3^ s^–1^.

At 753 K and 0.67 bar, Walker and co-workers^9,10^ measured relative 2-methylpropene yields for the

R1a $$i\text{‐}C_{4}H_{9}^{•}+O_{2}\to i\text{‐}C_{4}H_{8}+{\mathrm{HO}}_{2}^{•}\mathrm{and}$$

R2a $$t\text{‐}C_{4}H_{9}^{•}+O_{2}\to i\text{‐}C_{4}H_{8}+{\mathrm{HO}}_{2}^{•}$$

reactions with gas chromatography and determined *k*_1a_ = (1.1 ± 0.6) × 10^–13^ cm^3^ s^–1^ and *k*_2a_ = (7.1 ± 5.0) × 10^–13^ cm^3^ s^–1^, respectively. However, because the Arrhenius parameters of the reference reactions were not known accurately at the time, the uncertainties in the rate coefficients they report are quite large. Slagle et al.^11^ used 193 nm laser-photolysis (LP) to produce *t*-C_4_H_9_^•^ radicals from 4,4-dimethylpent-1-ene and monitored their decay in real time using PIMS at around 5 Torr between the 550 and 580 K. They observed multiexponential *t*-C_4_H_9_^•^ decays, indicating that *t*-C_4_H_9_^•^ + O_2_ ⇌ *t*-C_4_H_9_O_2_^•^ equilibration is taking place in their experiments.

A number of authors have investigated reactions R1 and R2 with computational methods. In an experiment-theory investigation, DeSain et al.^12^ used Cl-atoms to initiate *i*-C_4_H_10_ oxidation and monitored the formation of HO_2_^•^ in real time at temperatures between 298 and 693 K. Master-equation (ME) calculations were used to reproduce and explain the experimental observations. Miyoshi et al.^13^ used variational transition-state theory (VTST) and ME methods to investigate the kinetics of R2. Xing et al.^3^ have presented the most thorough computational investigation of R1. Moore et al.^14^ investigated the energetics of R2 at a very high level of theory, and also provide a helpful review of past works. No kinetic calculations were performed. Our group has published an experiment-theory study for R2,^5^ and here we seek to build and improve on this work.

In the present study, we performed LP-PIMS measurements to determine the rate coefficient of R1 as a function of temperature and pressure in He and N_2_ bath gases. New kinetic data are also reported for R2 by extending the measurements of our previous work to higher temperatures and pressures, as well performing measurements in N_2_ bath gas. In the previous work, we had incorrectly read the temperature in the reactor for the measurements that we report were performed at 420 and 494 K. We rectify this mistake here by redoing these measurements. In addition to the experimental work, we present ME models for both R1 and R2. In constructing these models, we leverage data reported in this work and past studies to optimize the values of key parameters in the models.

## Experimental Section

An overview of the experimental setup is given in a previous publication,^15^ so only the details pertinent to the present work are presented here. We performed experiments in tubular laminar-flow reactors made of stainless steel (halocarbon-wax coating) or quartz (boric-oxide or polydimethylsiloxane coating). The purpose of the coatings is to make the reactor walls as inert as possible to prevent the reactive loss of the radical under study on the walls. Reactors with inner diameters of 1.70 cm were used to perform low-pressure measurements (0.4–2 Torr), whereas reactors with diameters of 0.80 and 0.85 cm were used for measurements at slightly higher pressures (2–12 Torr). Both He and N_2_ bath gases were utilized.

For R1 and R2, the experiments covered the temperature ranges 220–550 K and 298–650 K, respectively. We could not extend the measurements of *k*_1_ beyond 550 K because at higher temperatures the unimolecular decomposition of *i*-C_4_H_9_^•^ becomes the dominant loss channel.^16^ For *t*-C_4_H_9_^•^, it was not possible to perform measurements above 650 K as the signal-to-noise ratio (SNR) became much lower. This is most likely due to the precursor beginning to thermally decompose at the experimental timescale. Between 220 and 370 K, the temperature of the reactors were controlled with a cooling/heating mantle that had methanol (220–298 K) or distilled water (298–370 K) circulating in it. Resistive coils wrapped around the reactors were used to heat the reactors to up to 650 K.

The radicals were homogeneously produced along the reactor by photolyzing suitable precursors with 193 or 248 nm photons. To produce *i*-C_4_H_9_^•^, we used 4-methylpentan-2-one (193 nm) or 1-iodo-2-methylpropane (248 nm), whereas for *t*-C_4_H_9_^•^ production we used 3,3-dimethylbutan-2-one (193 nm) or 2-bromo-2-methylpropane (248 nm). Consistent results were obtained with different precursors. The reactors are roughly one meter in length, and gas-flow velocities of 4–6 m s^–1^ were used. To ensure each laser pulse photolyzed a fresh gas mixture, we operated the exciplex laser at 5 Hz. A small portion of the gas-mixture (2–20%) was continuously drawn through a hole on the side of the reactor to a vacuum chamber containing a quadrupole mass spectrometer. To prepare the radicals for detection, they were ionised with radiation from a resonance-discharge lamp. We used a chlorine lamp (8.9–9.1 eV) with a BaF_2_ window to ionize *i*-C_4_H_9_^•^ and either a chlorine lamp with a BaF_2_ or CaF_2_ window or a nitrogen lamp (7.1 eV) with an optical-quality-quartz window to ionize *t*-C_4_H_9_^•^. The purpose of the windows is to filter out lines higher in energy than the desired dominant line.

In a typical measurement to determine the bimolecular rate coefficient of either R1 or R2, we first measured the decay rate coefficient of the radical without added O_2_. This loss is mainly due to reactions with the reactor walls, and thus we call it the wall rate for short (*k*_w_). To obtain *k*_w_, we fitted the function

1 $$\left[R^{•}\right]=A+Be^{-k_{w}t}$$

to a kinetic trace (concentration–time profile) measured without added O_2_. Note that [R^•^] here is not the absolute radical concentration, but rather a value directly proportional to it. After determining *k*_w_, we added a known concentration of O_2_ into the reactor. The employed [O_2_] was always in large excess over the radical concentration so that pseudo-first-order conditions were realized. The bath gas (He or N_2_) always constituted the bulk of the flow (>95%). After obtaining a kinetic trace with added O_2_, we fitted the function

2 $$\left[R^{•}\right]=A+Be^{-k\prime t}$$

to it to determine the pseudo-first-order rate coefficient *k*′. The bimolecular rate coefficient *k* we sought to determine is related to *k*′ and *k*_w_ by the expression

3 $$k^{\prime }=k\left[O_{2}\right]+k_{w}$$

We typically determined *k*′ at four or five different [O_2_] values. In the end, we repeated the wall-rate measurement to ensure *k*_w_ had remained approximately constant. We then plotted *k*′ (wall rates included) as a function of [O_2_] and fitted a straight line to the data. The slope of such a fit gave *k*, whereas the intercept gave a value for the wall rate *k*_w_. This should closely agree with the measured *k*_w_ values if the experiment has been correctly performed. We report both values. Example bimolecular plots for R1 and R2 are shown in Figure 2. This figure also shows examples of kinetic traces with and without added O_2_.

**Figure 2:**
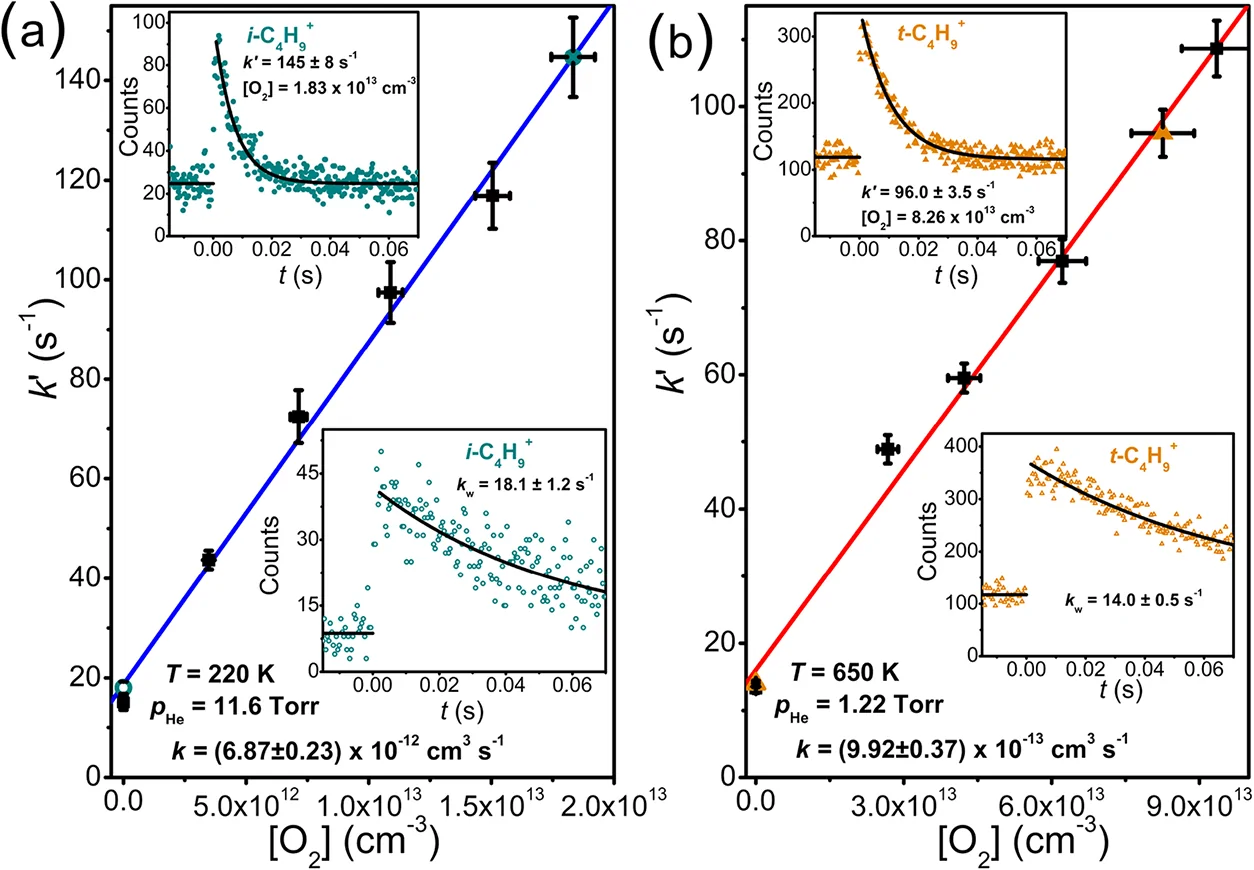
(a) Bimolecular plot for the *i*-C_4_H_9_ + O_2_ → Products reaction determined at 220 K and 11.6 Torr. (b) Bimolecular plot for the *t*-C_4_H_9_ + O_2_ → Products reaction determined at 650 K and 1.22 Torr. The colored *k*′ values correspond to similarly colored kinetic traces. The bottom-right and top-left traces show the measured *i*/*t*-C_4_H_9_^•^ signal in the absence and presence of added O_2_, respectively.

For both *i*- and *t*-C_4_H_9_^•^, we observed multiexponential-decay traces at around 550–600 K due to peroxyl-adduct dissociation back to the reactants. For R1 and R2, it is not possible to extract rate coefficients from such traces, but we nonetheless performed measurements in this temperature range because the traces can be used in ME trace fitting (see ME section).

To ensure the results were not affected by secondary chemistry, we systematically lowered the laser fluence and/or precursor concentration until we found that further adjustment no longer reduced the value of *k*_w_. Using butyl-radical concentrations low enough to suppress their self-reactions should also effectively suppress their reactions with other radicals like hydroxyl and hydroperoxyl—the expected products of R1 and R2. Furthermore, if such reactions were significant, this would have resulted in the traces having second-order character, but we found them to be strictly (pseudo)­first-order. In principle, hydroxyl, hydroperoxyl, and other reactive species could react with precursor molecules to initiate butyl-radical recycling. However, this would make the results dependent on the precursor and its concentration, and no such dependence was observed. Based on the employed laser fluences, the precursor concentrations, and the absorption cross sections of the precursor molecules, we estimate that the initial radical concentrations were [R^•^]_0_ < 2 × 10^11^ cm^–3^ in the experiments.

We estimate the overall uncertainty of the reported *k*_1_ and *k*_2_ values to be around ± 20%. This mostly arises from fitting and flow-rate-measurement uncertainties.

## Computational

### Quantum Chemistry

Stationary-point geometries were sought and optimized at the MN15/Def2TZVP level of theory.^17,18^ The same level of theory was used to determine harmonic frequencies and hindered-rotor and inversion potentials. A composite scheme was used for single-point energies. Initially, we computed ROHF-CCSD­(T) energies utilizing the cc-pVDZ, cc-pVTZ, and cc-pVQZ basis sets.^19,20^ The Hartree–Fock (HF) energies were extrapolated to the complete-basis-set (CBS) limit using the relation^21^

4 $$E_{\mathrm{HF}}\left(X\right)=E_{\mathrm{HF},\infty }+B_{\mathrm{HF}}e^{-\alpha_{\mathrm{HF}}X}$$

and for the slower converging correlation energy, we used the relation^22^

5 $$E_{\mathrm{corr}}\left(X\right)=E_{\mathrm{corr},\infty }+B_{\mathrm{corr}}X^{-\alpha_{\mathrm{corr}}}$$

Here *X* = 2, 3, or 4 stand for the cc-pVDZ, cc-pVTZ, and cc-pVQZ basis sets, respectively. In both cases, a system of three equations is solved to obtain the CBS-energy. All the above calculations were run with Gaussian 16.^23^ We further refined the results by calculating an additive Q-correction term

$$\Delta_{Q}E=\Delta E\left[\mathrm{ROHF}-\mathrm{CCSDT}\left(Q\right)/6\text{‐}31G^{*}\right]-\Delta E\left[\mathrm{ROHF}-\mathrm{CCSD}\left(T\right)/6\text{‐}31G^{*}\right]$$

for all the species. These calculations were run with the MRCC program.^24,25^ For the ROHF-CCSDT­(Q) energy, we opted to use the B-variant.^26^ Calculating Δ_Q_ *E* is particularly useful in cases where multireference effects are significant but not overwhelming. For the *t*-C_4_H_9_^•^ + O_2_ system, Moore et al.^14^ have computed additional correction terms (core-correlation, diagonal Born–Oppenheimer, and relativistic corrections), and we adopt their values to further improve our single-point energies. However, these three corrections are very small—usually well below 1 kJ mol^–1^ and often partially cancel each other out—so we chose not evaluate them for the *i*-C_4_H_9_^•^ + O_2_ system.

### Master Equation

All ME simulations were run with MESMER 7.1.^27^ MESMER is a one-dimensional ME code and so does not explicitly treat angular-momentum effects, but if experimental data are provided for parameter optimization (like in this work), some angular-momentum effects are implicitly captured. The chemical ME, as implemented in MESMER, describes a system of interconnected wells (species), where each well is divided into energy grains. The microcanonical rate coefficient *k*(*E*) is needed to compute the transition probability from the grain in one well to a grain in an adjacent well, and this is calculated with the RRKM expression

$$k\left(E\right)=\frac{N^{‡}\left(E\right)}{h\rho \left(E\right)}.$$ 6

Here *N* ^‡^(*E*) is the state sum of the transition state (TS) and ρ­(*E*) is the reactant state density. When quantum-chemistry calculations found a clear saddle-point for a reaction, we placed the TS there and used its rovibrational properties to determine *N* ^‡^(*E*). This obviously cannot be done for barrierless reactions (because there is no saddle-point), and to deal with these cases, we used the inverse Laplace transform (ILT) method.^28,29^ This method is natively implemented in MESMER and requires the user to provide a modified-Arrhenius-equation parametrization

7 $$k\left(T\right)=A{\left(\frac{T}{300K}\right)}^{m}e^{-E_{a}\prime /\mathrm{RT}}$$

of the canonical rate coefficient. Since *k*(*T*) can be expressed as a Laplace transform of *N* ^‡^(*E*), this relation can be inverted to obtain *N* ^‡^(*E*) as an ILT of *k*(*T*). For barrierless reactions, it is typical to set the exponential parameter *E*_a_^′^ to zero, and this was also done in this work. We obtained the other parameters through fitting to experiment (discussed in a later paragraph).

To obtain the transition probability from one grain to another within the same well, we used the traditional single-exponential-down model with the temperature-dependent parameter

8 $$\langle \Delta E\rangle_{\mathrm{down}}=\langle \Delta E\rangle_{\mathrm{down,}\mathrm{300 K}}{\left(\frac{T}{300K}\right)}^{n}$$

Here ⟨Δ*E*⟩_down,300 K_ is a parameter that describes the average energy transferred downward by collisions at 300 K, and *n* is a parameter used to capture the temperature dependence of this process. Both parameters are fittable. Lennard-Jones (LJ) interaction potentials were used to compute collision frequencies. For He and N_2_, we used literature LJ parameters,^30^ and for the butyl-peroxyl adducts (*i*/*t*-Int1), we estimated them with the online resources of RMG.^31^ The used values are listed in Table 1.

**Table 1:** Lennard-Jones Parameters Used in the Master-Equation Simulations

species	ϵ_LJ_ (K)	σ_LJ_ (Å)
He	10.22	2.551
N_2_	71.4	3.798
*i*-Int1	440	5.76
*t*-Int1	443	5.75

To partially capture anharmonic effects for vibrational modes, we scaled the harmonic frequencies we obtained from the quantum-chemistry calculations by 0.976.^32^ Several of the species considered have inversion potentials, and these were treated with the 1D-Fourier-grid method implemented in MESMER. This is a one-dimensional (uncoupled) approach. For species with hindered rotors, we applied the method of Gang et al.^33^ This method, in principle, fully treats the coupling between internal rotors and between external and internal rotors at the classical level. However, in this work we specified the multidimensional-rotor potential as a simple sum of 1D-rotor potentials (relaxed scans), so potential-energy coupling is not explicitly treated.

MESMER is equipped with tools that facilitate the use of experimental data to optimize parameters that are difficult and/or tedious to estimate with computational methods. This optimization can be done by minimizing differences between experimental and simulated rate coefficients (obtained from Bartis-Widom analysis^34,35^ ), or more recently, minimizing differences between experimental and simulated kinetic traces.^36^ The latter approach has a couple of advantages: it is a more direct comparison between experimental and computational data and can be used in cases where rate coefficients are difficult or impossible to extract from experimental traces. Trace fitting also remains valid in situations where the assumptions behind Bartis-Widom analysis fail. Here we opt to use trace fitting because it enables us to make use of multiexponential trace data. The parameters we optimized for both R1 and R2 were the modified Arrhenius parameters *A* and *m* (see eq 11) of the canonical R + O_2_ → RO_2_ recombination rate coefficients and the collisional-energy-transfer (CET) parameters ⟨Δ*E*⟩_down,300 K_ and *n*. ⟨Δ*E*⟩_down,300 K_ was optimized for both He and N_2_, but *n* was assumed to be the same for both gases. The reason for this choice was that we did not have enough temperature-dependent kinetic data in N_2_ to confidently fit it. For R2, we also optimized the relative energy of the concerted-elimination TS (TS1P1, see Figure 1b).

For R2, there is a fair amount of kinetic data published by other authors^8,11^ that we wanted to use in the fitting process. However, because we do not have access to their raw trace data, we chose to recreate their traces using the rate coefficients and fitting parameters they report. For consistency, we created traces in a similar manner from our data. To these ”signals”, we added noise that is comparable to that present in our measurements. We chose this approach over rate-coefficient fitting to take advantage of the multiexponential kinetic traces obtained in this work and by Slagle et al.^11^ Although the kinetic traces they recorded are presumably valid, the rate coefficients Slagle and Knyazev^37^ extracted from them are unphysical because the kinetic mechanism they utilized in the fits does not consider the well-skipping^38^ *t*-C_4_H_9_^•^ + O_2_ → *i*-C_4_H_8_ + HO_2_^•^ reaction (such reactions were not well understood at the time). When the well-skipping reaction is considered, the overall kinetic mechanism is

9 $$t\text{‐}C_{4}H_{9}^{•}+O_{2}\overset{k_{f}}{\underset{k_{b}}{⇌}}t\text{‐}C_{4}H_{9}O_{2}^{•}\overset{k_{p}\;}{\to }i\text{‐}C_{4}H_{8}+{\mathrm{HO}}_{2}^{•}$$

10 $$t\text{‐}C_{4}H_{9}^{•}+O_{2}\overset{k_{\mathrm{ws}}}{\to }i\text{‐}C_{4}H_{8}+{\mathrm{HO}}_{2}^{•}$$

and the function that describes *t*-C_4_H_9_^•^ decay is

11 $$\left[t\text{‐}C_{4}H_{9}^{•}\right]=Ae^{-\lambda_{1}t}+Be^{-\lambda_{2}t}$$

However, *A* and *B* are not independent parameters since [*t*-C_4_H_9_^•^]_*t*=0_ = *A* + *B*. Thus, it is not possible to extract the four phenomenological rate coefficients from the trace data since the fitting function has only three independent parameters.

We only optimized the CET parameters for the peroxyl adducts (*i*/*t*-Int1); for the QOOH species, we used the expressions derived by Hansen et al.^39^ for (CH_3_)_2_C­(OOH)­CH_2_^•^ (the QOOH present in the R2 reaction system):

12 $$\langle \Delta E\rangle_{\mathrm{down}}^{\left(\mathrm{He}\right)}=60{\left(\frac{T}{300K}\right)}^{0.62}{\mathrm{cm}}^{-1}$$

13 $$\langle \Delta E\rangle_{\mathrm{down}}^{\left(N_{2}\right)}=150{\left(\frac{T}{300K}\right)}^{0.60}{\mathrm{cm}}^{-1}$$

We also adopt their expression for the canonical rate coefficient of the

$$({\mathrm{CH}}_{3}{)}_{2}C\left(\mathrm{OOH}\right){\mathrm{CH}}_{2}^{•}\to {\left({\mathrm{CH}}_{3}\right)}_{2}\overset{®}{\mathrm{COC}}H_{2}+{\mathrm{OH}}^{•}$$

­(2,2-dimethyloxirane + hydroxyl) reaction, which is

14 $$k\left(T\right)=4.95\cdot 10^{11}{\left(\frac{T}{300K}\right)}^{0.894}e^{-4587K/T}s^{-1}$$

This was converted to *k*(*E*) with the ILT technique.

The grain-sizes of the R1 and R2 simulations were 25 and 40 cm^–1^ respectively. We set the energy threshold to be 25*k*_B_ *T* above the highest-energy stationary point. Eckart-tunnelling corrections were computed for all reactions involving the transfer of a hydrogen atom. About 380 *i*-C_4_H_9_^•^ and 70 *t*-C_4_H_9_^•^ traces were utilized to perform the fits. These traces are included in the ME input files we provide in the Supporting Information.

## Results and Discussion

### Quantum Chemistry

The results of our quantum-chemistry calculations are tabulated in Tables 2 and 3. We depict the results graphically in Figure 1, and this figure also introduces the notation used for the chemical species. Note that we use the same identifiers for analogous species in both reaction systems. When necessary, the prefixes *i* or *t* are used to distinguish analogous structures (*i*-Int1 and *t*-Int1, for example). Note that the products P1 and P2 are the same for R1 and R2. We expect our results to achieve chemical accuracy (4 kJ mol^–1^) for the species with small Q corrections (less than 1 kJ mol^–1^). When it is larger than this, it is harder to assess the energy uncertainty as the species might have non-negligible multireference nature.

**Table 2:** Relative Stationary-Point Energies for the *i*-C_4_H_9_^•^ + O_2_ Reaction System^a^

species	σ_ext_ σ_int_/*m*_opt_ ^b^	ΔZPE^c^	ROHF-CCSD(T)/CBS^d^	Δ_Q_ *E* ^e^	total^f^	Xing et al.^3^
R	1 × 18/1, 2 × 1/1	0	0 (0.012,0.017)	0	0	0
P1		4.69	–78.47 (0.010, 0.032)	0.48	–77.99	
P2		4.12	–173.18 (0.010, 0.013)	2.84	–170.34	
P3		8.92	–146.53 (0.010, 0.013)	2.86	–143.68	
Int1	1 × 9/1	20.85	–141.09 (0.024)	0.92	–140.17	–143.8
Int2	1 × 18/1	13.15	–94.70 (0.014)	1.12	–93.58	
Int3	1 × 6/2	13.64	–71.77 (0.013)	1.41	–70.36	
TS12	1 × 9/2	4.75	–12.17 (0.023)	–0.10	–12.28	–16.94
TS13a	1 × 3/2	5.25	–31.87 (0.023)	–0.04	–31.91	
TS13b	1 × 3/2	5.17	–32.20 (0.023)	0.01	–32.20	–38.20
TS1P1	1 × 9/1	3.46	–6.52 (0.027)	–2.07	–8.59	–10.88
TS2P1	1 × 9/1	8.74	–29.82 (0.032)	–1.58	–31.4	
TS2P2	1 × 9/1	6.08	–52.89 (0.023)	–2.94	–55.83	
TS3P3a	1 × 3/2	10.27	9.48 (0.029)	–3.11	6.37	
TS3P3b	1 × 3/2	10.05	8.55 (0.029)	–3.16	5.40	

a Total and CCSD­(T) energies are zero-point-energy (ZPE) inclusive. All energies are in kJ mol^–1^.

b Here σ_ext_, σ_int_, and *m*_opt_ are the external-rotation, internal-rotation/inversion, and optical symmetry numbers, respectively.

c MN15/Def2-TZVP

d Values in the parentheses are T1 diagnostics.

e ΔROHF-CCSDT­(Q)/6–31G*– ΔROHF-CCSD­(T)/6–31G*­(B-variant^26^)

f Sum of the preceding two columns.

**Table 3:** Relative Stationary-Point Energies for the *t*-C_4_H_9_^•^ + O_2_ Reaction System^a^

species	σ_ext_ σ_int_/*m*_opt_ ^b^	ΔZPE^c^	ROHF-CCSD(T)/CBS^d^	Δ_Q_ *E* ^e^	corr^f^	total^g^	Moore et al.^14^
R	3 × 54/1, 2 × 1/1	0	0 (0.013, 0.017)	0	0	0	0
P1		5.07	–56.01 (0.010, 0.032)	0.63	0.03	–55.34	–57.32
P2		4.50	–150.73 (0.010, 0.013)	2.99	–0.18	–147.91	–158.99
Int1	1 × 81/1	17.91	–149.71 (0.024)	1.14	∼0	–148.58	–150.21
Int2	1 × 13.5/1^h^	10.54	–74.40 (0.013)	1.60	0.45	–72.36	–79.91
TS12	1 × 9/2	1.76	6.15 (0.025)	–0.05	0.65	6.74	0.84
TS1P1	1 × 9/1	2.26	–21.22 (0.028)	–1.74	0.44	–22.52	–23.43
TS2P1	1 × 9/1	8.24	–7.95 (0.024)	–2.76	0.59	–10.1	–2.09
TS2P2	1 × 9/1	5.72	–23.02 (0.024)	–3.66	0.71	–25.97	–29.71

a Total and CCSD­(T) energies are zero-point-energy (ZPE) inclusive. All energies are in kJ mol^–1^.

b Here σ_ext_, σ_int_, and *m*_opt_ are the external-rotation, internal-rotation/inversion, and optical symmetry numbers, respectively.

c MN15/Def2-TZVP

d Values in the parentheses are T1 diagnostics.

e ΔROHF-CCSDT­(Q)/6–31G*- ΔROHF-CCSD­(T)/6–31G*­(B-variant^26^)

f The sum of relativistic, core-correlation, and diagonal-Born–Oppenheimer corrections (from Moore et al.^14^).

g Sum of the preceding three columns.

h 3 × 3 × 1.5 = 13.5. See the SI of Hansen et al.^39^ (Figure S13) for a discussion regarding the symmetry number of this QOOH species.

For R1, a thorough comparison between our results and those of Xing et al.^3^ is not possible as they do not explicitly list their stationary-point energies, but some energies are mentioned in the text. As shown in Table 2, their CCSD­(T)-F12a/jun-cc-pVTZ//M05–2X/jun-cc-pVTZ energies are systematically a few kJ mol^–1^ lower than ours, but most likely the energies agree within the expected uncertainties of both sets of calculations. They make no mention of finding multiple saddle-points for some of the reactions (TS13a/TS13b and TS3P3a/TS3P3b), so it is not clear if these saddle-points were identified and/or considered in the subsequent ME simulations.

For R2, the agreement with Moore et al.^14^ is good for some species, but for several species the disagreement is more than few kJ mol^–1^. The most obvious explanation for the disagreement is that we performed single-point-energy calculations with MN15/Def2TZVP geometries, whereas they used CCSD­(T)/ANO0 structures. Fortunately, agreement is good for the energies of the two species that the ME model is sensitive to Int1 and TS1P1. Nevertheless, we were surprised to find such large disagreement for stable species like Int2 and P2, but as the Q-corrections suggest, these calculations might not be as straightforward as one could naively think.

### Experiments and Trace Fits

We summarize the results of our experiments in Tables S1, S2 and S3 in the Supporting Information (SI). To run the trace fits, we used the present results for R1, and for R2, we used the present results together with those from our previous study,^5^ Dilger et al.,^8^ and Slagle et al.^11^ The results from Bayes and co-workers^4,7^ were not included in the fits as we could not unambiguously assign a pressure to their data points (*k*′ values were measured at different total pressures). The optimized ME parameters returned by the trace fits are listed in Table 4 with their 1-σ uncertainties. Example comparisons between measured traces and those output by the optimized ME model for R1 are shown in Figure 3, and good agreement is observed. Note that in subfigure (b) the signal decay is multiexponential.

**Figure 3:**
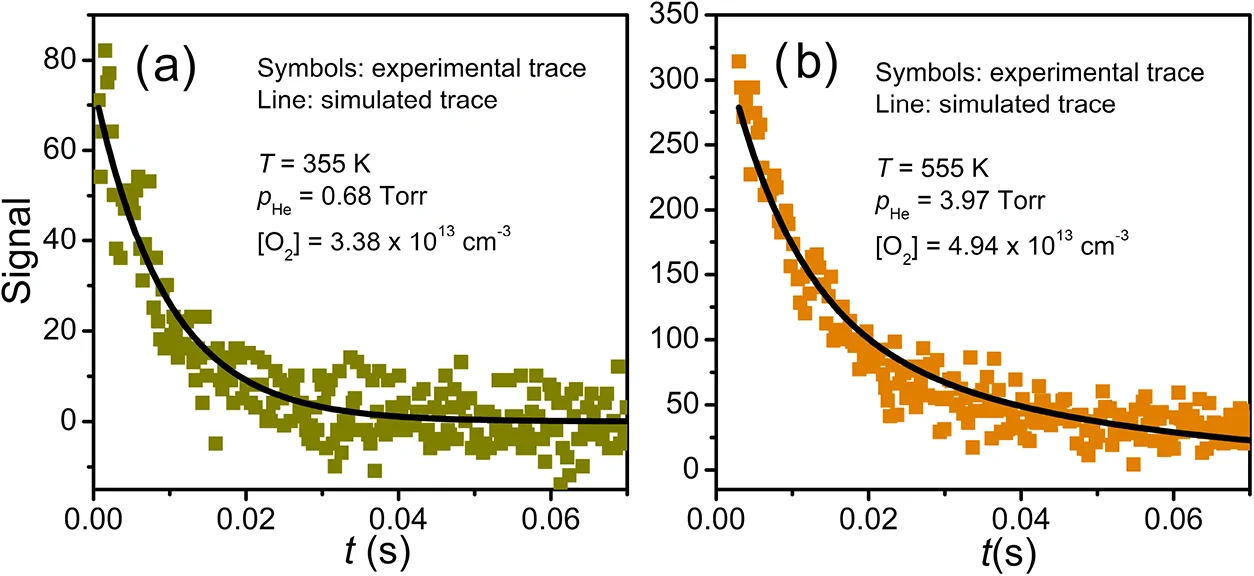
Measured and simulated *i*-C_4_H_9_^•^ traces compared at (a) 355 K and (b) 555 K.

**Table 4:** Optimized Master-Equation Parameters Returned by the Trace Fits for the *i*-C_4_H_9_^•^ + O_2_ and *t*-C_4_H_9_^•^ + O_2_ Reaction Systems

parameter	value
*i*-C_4_H_9_^•^ + O_2_	
*A* (10^–12^ cm^3^ s^–1^)	4.57 ± 0.43
*m*	–0.995 ± 0.284
*i*-Int1:⟨Δ*E*⟩_down,300 K_^(He)^ (cm^–1^)	57.1 ± 26.5
*i*-Int1:⟨Δ*E*⟩_down, 300 K_^(N_2_)^ (cm^–1^)	167 ± 124
*i*-Int1: *n*	2.60 ± 0.85
*t*-C_4_H_9_^•^ + O_2_	
*A*(10^–12^ cm^3^ s^–1^)	18.5 ± 1.3
*m*	–1.46 ± 0.22
*t*-Int1: ⟨Δ*E*⟩_down,300 K_^(He)^ (cm^–1^)	176 ± 73
*t*-Int1: ⟨Δ*E*⟩_down,300 K_^(N_2_)^ (cm^–1^)	352 ± 184
*t*-Int1: *n*	1.56 ± 0.91
Δ*E*[TS1P1] (kJ mol^–1^)	–28.60 ± 0.37

As with other alkyl radical + O_2_ reactions, the kinetics of R1 and R2 can be divided into low- (LT), intermediate-, and high-temperature (HT) regimes.^40,41^ The exact temperature ranges of these regimes depend weakly on pressure and [O_2_], but for R1 and R2, they are roughly *T* < 550 K (LT), 550–650 K (intermediate temperature), and *T* > 650 K (HT). In the LT regime, single-exponential decays are observed (if O_2_ is in excess), and the observed rate coefficient is a simple sum of the adduct- and bimolecular-product-forming phenomenological rate coefficients. Peroxyl-adduct dissociation back to R^•^ + O_2_ becomes significant in the intermediate-temperature regime, and multiexponential decays are observed. Single-exponential decays re-emerge in the HT regime where the R^•^ + O_2_ ⇌ RO_2_^•^ equilibrium overwhelmingly favors the reactants and is established almost immediately, and the butyl radicals decay by a simple pre-equilibrium mechanism:

15 $$R^{•}+O_{2}⇌{\mathrm{RO}}_{2}^{•}\to \mathrm{Products}$$

16 $$R^{•}+O_{2}\to \mathrm{Products}\left(\mathrm{well}‐\mathrm{skipping}\right)$$

The HT rate coefficient is virtually pressure independent, even though the individual elementary rate coefficients it is a function of are not.^41^

For R2, P1 is essentially the only product channel, and applying the pre-equilibrium approximation returns

17 $$k_{2}=k_{R\to P1}+\frac{k_{R\to \mathrm{Int}1}k_{\mathrm{Int}1\to P1}}{k_{\mathrm{Int}1\to R}+k_{\mathrm{Int}1\to P1}}$$

for the HT rate coefficient. However, a simpler approach exists to compare experimental and simulated results. If a single-exponential decay exists, there will be a chemically significant eigenvalue (CSE) that describes it. The relationship between this CSE and the experimentally determined rate coefficient is simply

18 $$k=-\frac{\lambda_{i}}{\left[O_{2}\right]}$$

#### Low-Temperature Rate Coefficient

The LT experimental data are plotted as a function of pressure at selected temperatures in Figure 4a,b together with the falloff curves produced by our optimized ME models. At 200–223 K, both R1 and R2 appear to be close to the high-pressure limit (HPL) at around 0.001–0.01 bar, whereas at 495 K, the reactions are clearly in the falloff in the same pressure range. As can be seen, the optimized models are able to reproduce the temperature and pressure dependent data reasonably well, although not perfectly—especially for R1. The disagreement for R1 is the largest at 220 K, where the experimental values are consistently larger than simulated ones. This suggests either that eq 11 is not flexible enough to describe the temperature dependence of the canonical rate coefficient, or that there is a minor issue in the 220 K measurements. That said, even at 220 K the model-experiment disagreement is within experimental uncertainty.

**Figure 4:**
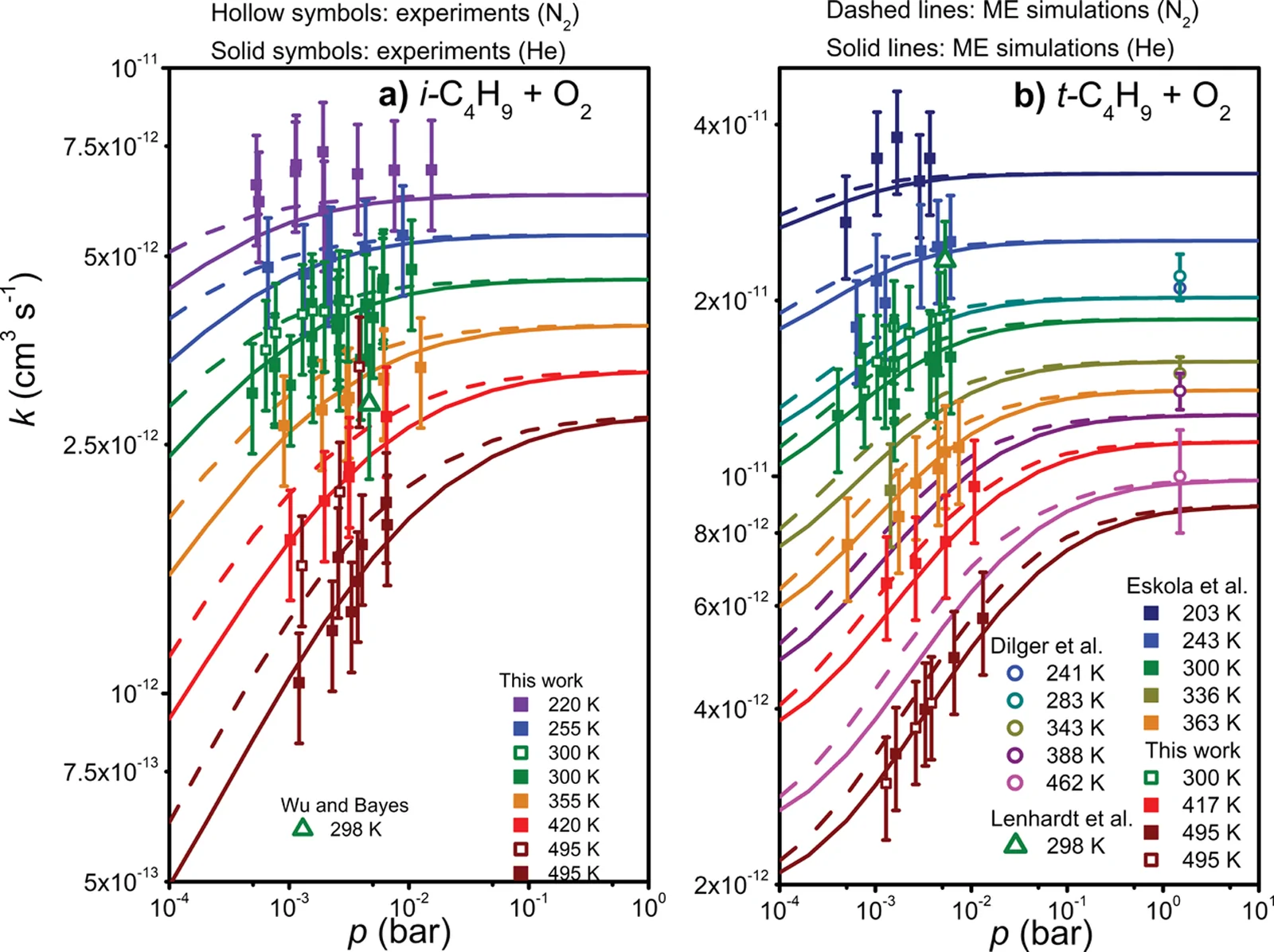
Measured and simulated pressure dependence of the rate coefficient of reaction (a) *i*-C_4_H_9_^•^ + O_2_ → Products and **(**b) *t*-C_4_H_9_^•^ + O_2_ → Products plotted at selected temperatures. The data from Eskola et al.^5^ and Dilger et al.^8^ were included in the trace fits, but the data from Bayes and co-workers were not.^4,7^

A somewhat concerning result is that the uncertainties of the CET parameters are quite large. This should not be due to the lack of data, nor due to overfitting as the uncertainties on the Arrhenius parameters are quite small. We suspect this is because much of the experimental data are in pressure ranges where the ME models are not very sensitive to the value of the CET parameters. This can be deduced by comparing the He and N_2_ falloff curves. For R1, the data below 400 K are sufficiently close to the HPL that the pressure effect is quite weak, whereas for R2, well-skipping is significant even at low temperatures, and the CET parameters mostly affect the branching between well-skipping and peroxyl-adduct-forming reactions rather than the value of the overall rate coefficient. Another contributing factor for R1 is that the signals we measured were often quite noisy (low SNRs), which naturally increases the fitting uncertainties. PIMS is not as sensitive to detecting *i*-C_4_H_9_^•^ as it is to *t*-C_4_H_9_^•^, and we were only able to obtain acceptable signals by using new or recently cleaned VUV-quality BaF_2_ windows with a chlorine lamp. Despite the large fitting uncertainties, the values of the CET parameters are reasonable and are able to reproduce the observed pressure dependencies. One might find the factor of 3 difference between the ⟨Δ*E*⟩_down,300 K_ values of *i*- and *t*-Int1 surprising, but comparable differences were observed in our investigation of the unimolecular decomposition reactions of butyl radicals.^16^ That said, given the large uncertainties in the ⟨Δ*E*⟩_down,300 K_ parameters of *i*- and *t*-Int1, it is unclear if the differences are in fact a factor of 3, and thus we are hesitant to speculate what might explain the observed differences.

The rate-coefficient values Bayes and co-workers^4,7^ determined for R1 and R2 at room temperature are 25% smaller and 25% larger, respectively, but the results from both groups agree within stated uncertainties. They observed no pressure dependence for R1 (2.3–3.5 Torr) and R2 (1–4 Torr), but we believe this to be because the covered pressure ranges are too narrow. Going from the low to the high end of the ranges only result in rate-coefficient increases that are smaller than measurement uncertainties. Interestingly, the disagreement between the current results and those of Bayes and co-workers is not systematic. Without having a detailed knowledge of their experiments, it is difficult to speculate as to source of the disagreement. The experimental setups are very similar, with the only qualitative difference being that they employ flash photolysis rather than laser photolysis for radical production. They also opted to use a xenon lamp (8.4 eV) with a sapphire window for ionization. We found this lamp to produce good-quality signals for *t*-C_4_H_9_^•^, but not for *i*-C_4_H_9_^•^.

#### High-Temperature Rate Coefficient

We illustrate in Figure 5 for R2 how the experimentally determined rate coefficient corresponds λ_2_ and λ_1_ in the LT and HT regimes, respectively. In the intermediate temperature range (550–650 K), λ_1_ and λ_2_ are comparable in magnitude, and the observed and simulated decays are multiexponential. This figure also demonstrates that the steady-state expression for the HT rate coefficient (eq 21) is essentially identical to −λ_1_/[O_2_], as well as that the HT rate coefficient is virtually pressure independent.

**Figure 5:**
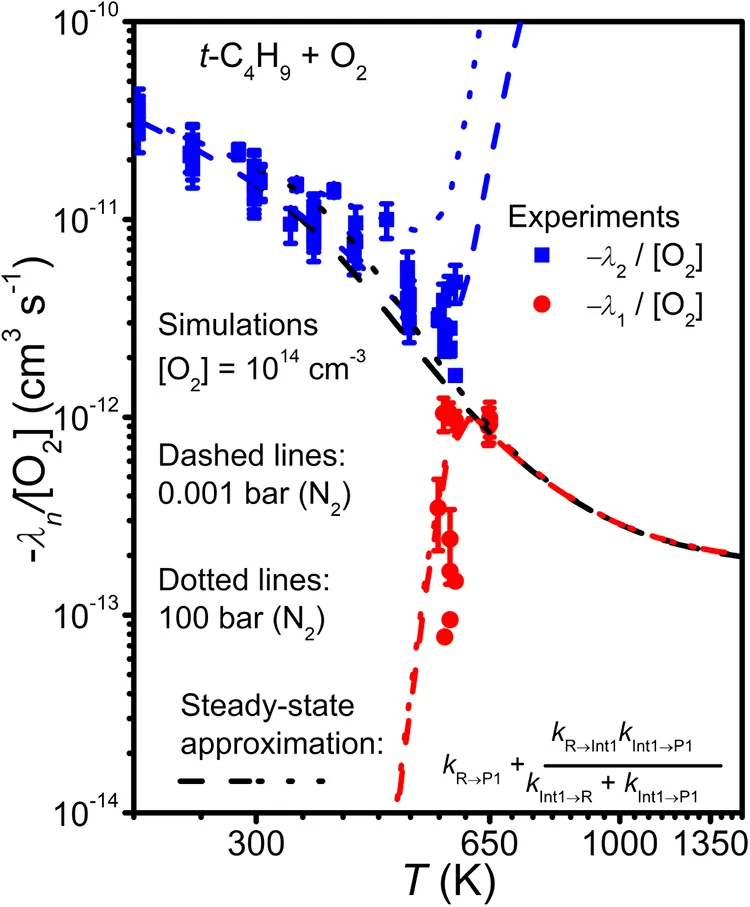
Simulated and experimental^5,8,37^ – λ_1_/[O_2_] and – λ_2_/[O_2_] values for the *t*-C_4_H_9_^•^ + O_2_ → Products reaction plotted as a function of temperature.

For R2, the high-temperature rate coefficient is almost solely sensitive to the height of concerted-elimination barrier TS1P1. To reproduce the experimental data at around 650 K, we had to lower TS1P1 from −22.52 to −28.60 kJ mol, which is quite a large adjustment. It is not clear to us if such a large reduction is justified for TS1P1. The T1 diagnostic is not too large (0.028), Moore et al.^14^ found the CASSCF coefficient *C*_0_^2^ to be 0.94, and the Q correction is modest (−1.74 kJ mol). In fact, the only unusual thing Moore et al. found about TS1P1 is that the CCSDT­(Q)/B and CCSDT­(Q)/A energies differed by roughly 0.71 kJ mol, whereas for other species the differences are negligible. We were able to reproduce this finding, and furthermore, found a similar deviation for the equivalent structure for R1. So we are unsure if there is something nontrivial about the electronic-structure calculation of this species. In the end, we decided to adopt the adjustment in our final model in order to reproduce the high-temperature data. Further study is needed to adjudicate whether this is an experimental or computational problem—or some combination thereof.

Figure 6 shows the channel-specific rate coefficients and reaction yields of R1 and R2 as a function of temperature in 0.01 and 100 bar of N_2_. As can be seen, both the rate coefficients and reaction yields are pressure independent for all practical purposes. The channel-specific rate coefficients were determined by multiplying – λ_1_/[O_2_] with the yields of the bimolecular-product sinks (P1, P2, and/or P3), which in turn were determined by simulating the reaction systems long enough for all the population to be in the sinks. The results are shown alongside the results of Walker and co-workers^9,10^ for R1a and R2a. Strictly speaking, they did not perform direct kinetic measurements, but rather determined the rate coefficients of R1a and R2a relative to the *i*-C_4_H_9_^•^ → C_3_H_6_ + CH_3_^•^ and *t*-C_4_H_9_^•^ + H_2_ → *i*-C_4_H_10_ + H^•^ reactions, respectively. Although they report absolute rate coefficients, the values they used for the reference reactions are quite dated and based on a number of estimates and approximations. Here we recalibrate the results with more recent values for the reference reactions.^16,42^ The recalibrated values for R1a and R2a are roughly a factor of 2 smaller and a factor of 3–4 larger, respectively, than the original values.^9,10^ For both R1a and R2a, the recalibrated rate-coefficient values are about a factor of 2 larger than the corresponding values produced by our optimized ME models. The present models predict the chain-terminating conjugate-alkene channel (P1) to be dominant for R2 at all temperatures, but for R1, its yield is only about 30%; the hydroxy-producing chain-propagating channels make up the rest of the yield.

**Figure 6:**
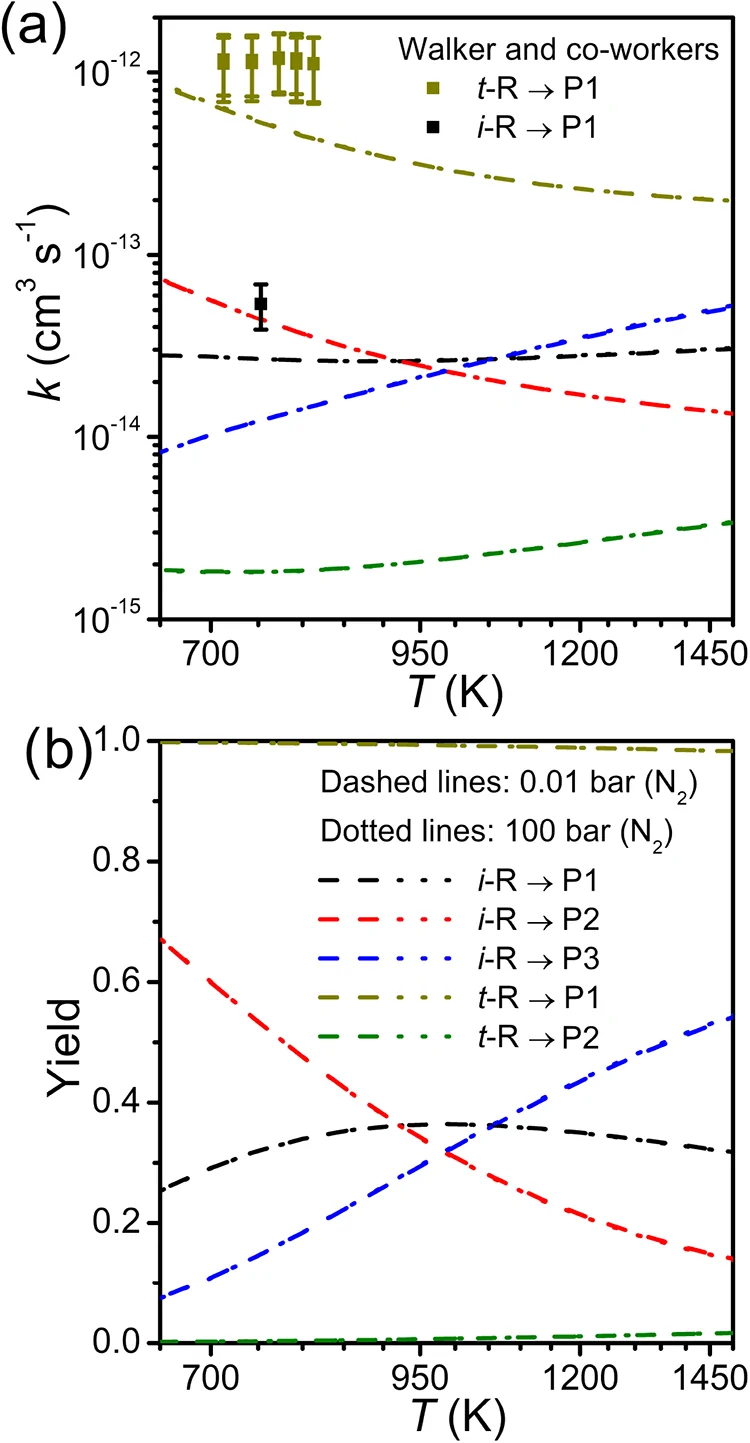
Simulated channel-specific rate coefficients (a) and reaction yields (b) for the *i*-C_4_H_9_^•^ + O_2_ → Products and *t*-C_4_H_9_^•^ + O_2_ → Products reactions plotted as a function of temperature in 0.01 and 100 bar of N_2_. Also shown are the experimental results of Walker and co-workers for the conjugate-alkene channels.^9,10^

### Canonical Rate Coefficients

We plot in Figure 7 the canonical R^•^ + O_2_ recombination rate coefficients obtained in this work together with literature values. For comparison purposes, we also plot such rate coefficients for related alkyl radicals. Some of these are based on variational TST methods,^3,6,13,43^ but others have been obtained from ILT fitting like the present results.^44,45^ The value we obtain for R1 is about 50% larger than the prediction of Xing et al.^3^ No uncertainty estimate is reported, but given that they used CASPT2 to characterize the minimum-energy path, a 50% or so uncertainty would not be unusual. For R2, the room-temperature prediction of Miyoshi agrees well with our result, but the results diverge at higher temperatures.

**Figure 7:**
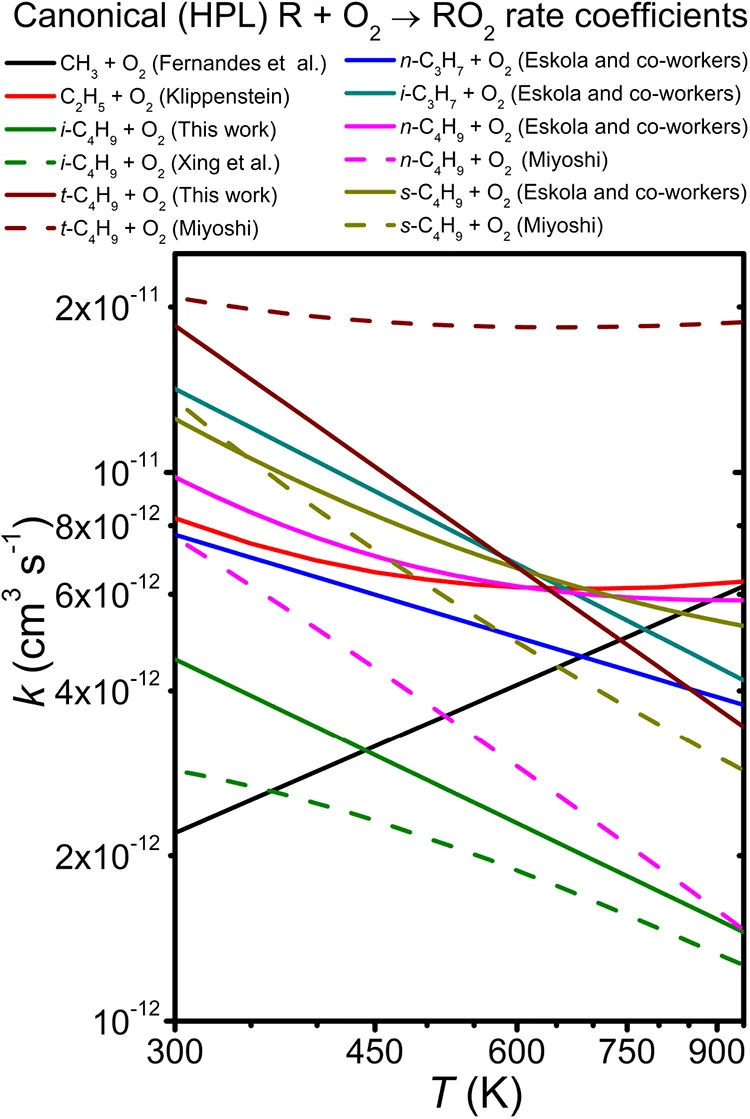
Canonical R^•^ + O_2_ rate coefficients for C1, C2, C3, and C4 alkyl radicals. The rate coefficients are from Fernandes et al.,^46^ Klippenstein,^43^ Eskola and co-workers,^6,40,44,45^ Miyoshi,^13^ and Xing et al.^3^

For several of the reactions, the variational calculations predict the temperature dependence of the canonical rate coefficient to shift from negative to positive at around 700 K. A limitation of the ILT method—when utilizing a single modified Arrhenius equation—is that it cannot predict such a change in temperature dependence. We also wish to point out that for the reactions for which the ILT method was used, the experimental data was obtained at temperatures below 750 K. Thus, we do no expect the ILT expression to extrapolate well to temperatures beyond 750 K. Fortunately, at elevated temperatures the kinetic bottleneck for bimolecular-product formation is typically not the loose entrance TS.

The results shown in Figure 7 indicate that methyl substitution of the radical center enhances reactivity (*t*-C_4_H_9_^•^ > *i*-C_3_H_7_^•^ > C_2_H_5_^•^ > CH_3_^•^), but making this substituent longer has a roughly neutral effect (C_2_H_5_^•^, *n*-C_3_H_7_^•^, and *n*-C_4_H_9_^•^ have similar reactivities, as do *i*-C_3_H_7_^•^, and *s*-C_4_H_9_^•^). Interestingly, it seems that making the hydrocarbon chain branched abates reactivity; *i*-C_4_H_9_^•^ has the lowest reactivity of all the C2, C3, and C4 alkyl radicals, and the measurements of Wu and Bayes^4^ show that the neo-pentyl radical reacts even slower with O_2_ than *i*-C_4_H_9_^•^. The reactivity of CH_3_^•^ is fairly unique here: it reacts the slowest at room temperature, but at elevated temperatures the reactivity becomes comparable to other alkyl radicals. It also is the only radical in this set that has positive temperature dependence across the whole temperature range.

### Comparison with Xing et al. Results

We plot in Figure S1 the ratio of the Xing et al.^3^ and our phenomenological rate coefficients for R1. Some lines end abruptly because CSEs and IEREs are no longer well separated (here we plotted rate coefficients as long as λ_*n*+1_^IERE^/λ_*n*_^CSE^ > 2). Much of the results are in the range 0.5–2, which is typical accuracy for kinetic calculations. An in-depth discussion regarding the differences is not possible as we do not know all the stationary-point energies in their model—or other implementation details such as symmetry numbers—but they do mention using ⟨Δ*E*⟩_down_^(Ar)^ = 300­(T/300 K)^0.85^ cm^–1^ for Int1, Int2, and Int3. This is roughly twice as large as the parameter we obtained for N_2_.

### QOOH + O_2_

To investigate the possible importance of second-O_2_ addition, we added into our ME models QOOH + O_2_ → Products reactions. Xing et al.^3^ computed the rate coefficient for the *i*-Int3 + O_2_ → Products reaction, and here we adopt their value for all the QOOH + O_2_ reactions. We treat these reactions as irreversible and estimate their microcanonical rate coefficients to be *k*(*E*) = *k*_Xing_(650 K)­[O_2_]. Although these approximations are quite crude, the purpose here is merely to obtain order-of-magnitude estimates for the total yields of the second-O_2_-addition reactions. Simulations were run at 650 K in 0.1 and 10 bar of N_2_ (*x*(O_2_) = 0.21), and the resulting concentration–time profiles are shown in Figure 8. For R2, hardly any products are formed by the *t*-Int2 + O_2_ → Products reaction, and it should be safe to ignore this pathway in combustion modeling. The analogous *i*-Int2 + O_2_ → Products reaction is more important for R1, but its yield is still less than 1% at 10 bar. However, at 10 bar our crude model predicts that the Int3 + O_2_ → Products reaction is the most important channel for R1, and this pathway certainly needs to be included in 2-methylpropane combustion models.

**Figure 8:**
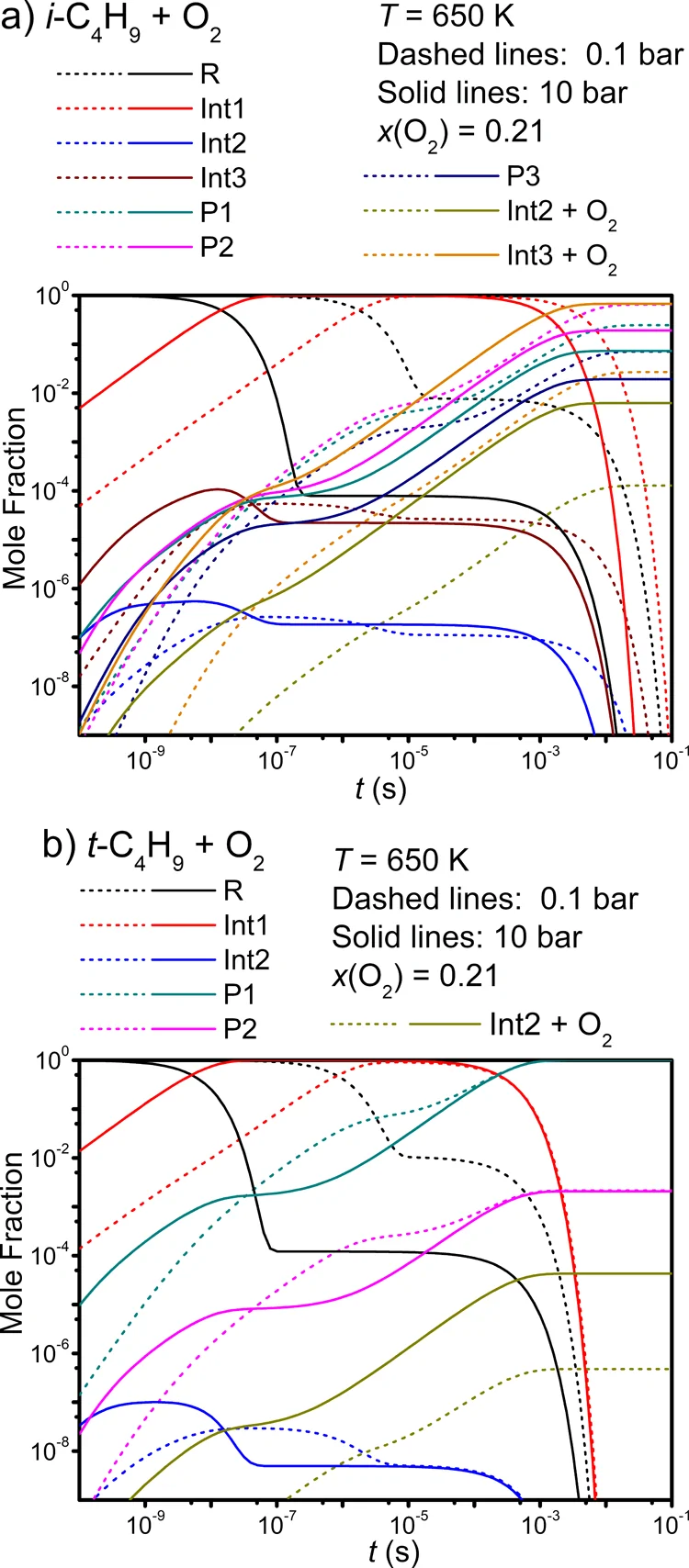
Concentration–time profiles produced by the (a) *i*-C_4_H_9_^•^ + O_2_ and (b) *t*-C_4_H_9_^•^ + O_2_ master-equation models at 650 K in 0.1 bar (dashed lines) and 10 bar (solid lines) of N_2_. QOOH + O_2_ → Products reactions have been added to the master-equation models in these simulations.

### Model Reduction

As the concentration–time profiles shown in Figure 8 suggest, the Int2 species are only present at small steady-state concentrations. Under typical combustion conditions, any Int2 formed will quickly react further to produce P1 and P2. Thus, to reduce the number of modeled species, one can apply the steady-state approximation (at the microcanonical level) to derive the following “effective state sums” for P1 and P2 product formation:

19 $$N_{P1,\mathrm{eff}}^{‡}=N_{\mathrm{TS}1P1}^{‡}+\frac{N_{\mathrm{TS}12}^{‡}N_{\mathrm{TS}2P1}^{‡}}{N_{\mathrm{TS}12}+N_{\mathrm{TS}2P1}^{‡}+N_{\mathrm{TS}2P2}^{‡}}$$

20 $$N_{P2,\mathrm{eff}}^{‡}=\frac{N_{\mathrm{TS}12}^{‡}N_{\mathrm{TS}2P2}^{‡}}{N_{\mathrm{TS}12}+N_{\mathrm{TS}2P1}^{‡}+N_{\mathrm{TS}2P2}^{‡}}$$

For R1, further simplification is possible. The barrier for Int1 ⇌ Int3 isomerization is low, and thus the reaction expected to readily equilibrate even in LTC. Thus, we merge these two species and consider an effective species with the state density

21 $$\rho_{13}\left(E\right)=\{\begin{array}{ll} \rho_{1}\left(E\right) & E<E_{\mathrm{Int}3}\\ \rho_{1}\left(E\right)+\rho_{3}\left(E\right) & E\geq E_{\mathrm{Int}3} \end{array}$$

That is, it is assumed that Int1 and Int3 are always in microcanonical equilibrium with each other. In Figures S2 and S3 in the SI, we show how the full and reduced models predict very similar concentration–time profiles for R, Int1, and the product species over a wide range of conditions. For R2, the profiles are essentially identical, but even for R1, the differences in product yields are are only 1–2% in the worst cases. The 1–2% difference is observed under high [O_2_] conditions, where the assumption that the steady-state concentration of *i*-Int2 is negligible is less good. Furthermore, the general uncertainties in the yield prediction are much larger than 2% due to uncertainties in stationary-point energies and other ME parameters. The good agreement between the full and reduced models at 650 K, *p*_N_2__ = 1 bar, and *x*(O_2_) = 0.21 is depicted graphically in Figure 9a,b.

**Figure 9:**
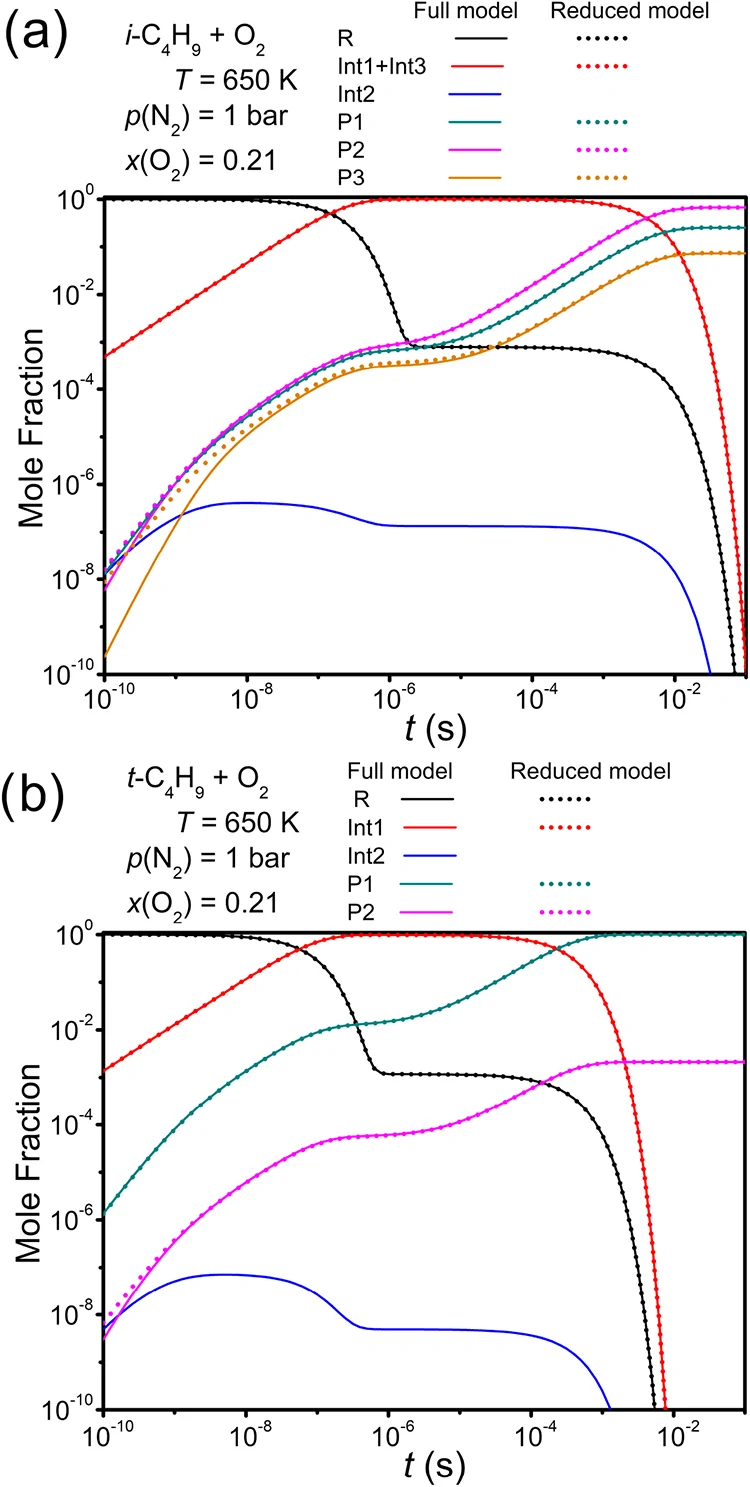
Concentration–time profiles produced by the full and reduced (a) *i*-C_4_H_9_^•^ + O_2_ and (b) *t*-C_4_H_9_^•^ + O_2_ master-equation models at 650 K in 1 bar of N_2_.

Reducing the models achieves two things.

(1) It significantly reduces the number of phenomenological rate coefficients that need to be incorporated in combustion models. R1 is reduced from 18 to 7 rate coefficients and R2 from 9 to 5.
(2) It extends the temperature and pressure ranges in which CSEs and IEREs are well separated, and thus the temperature and pressure ranges in which Bartis-Widom analysis can be used to determine phenomenological rate coefficients. In general, the shallower the wells in a model are, the lower the temperature and/or higher the pressure at which CSE-IERE overlap becomes an issue. Here we have eliminated all but the deepest well for both R1 and R2.

An obvious drawback of the reduction is that we no longer have a QOOH species that can initiate second-O_2_-addition chemistry, and as our testing showed, this is important for R1. Thus, we need to initiate this chemistry from the merged Int1/Int3-admixture species. Assuming Int1 and Int3 are perfectly equilibrated, the fraction of the Int1/Int3-admixture species that is in the Int3 well is

22 $$\left[\mathrm{Int}3\right]=\frac{K}{K+1}\left[\mathrm{Int}1/\mathrm{Int}3\right]$$

where *K* is the Int1 ⇌ Int3 equilibrium constant. Thus, any Int3 + O_2_ rate coefficient can be multiplied by *K*/(*K*+1) to get the corresponding Int1/Int3-admixture + O_2_ rate coefficient. We provide *K* as a function of temperature in the SI, as well as PLOG and Chebyshev parametrizations of rate coefficients produced by the reduced models.

## Conclusions

We performed laser-photolysis photoionization mass-spectrometry experiments to determine the rate coefficient between 2-methylpropan-1-yl and molecular oxygen. This is the first study in which this reaction’s temperature and pressure dependence has been probed. Similar measurements were performed for the 2-methylpropan-2-yl radical. The experimental work was complemented with quantum-chemistry calculations and master-equation simulations. After using the experimental data to optimize master-equation parameters related to collisional-energy transfer and barrierless reactions, the master-equation simulations were able to reproduce the experimental results. When possible, the current results were compared with past experimental and computational results. Simulations were performed over a wide range of conditions, and the results are parametrized in PLOG and Chebyshev formats to facilitate their use in combustion modeling. The 2-Methylpropan-2-yl + O_2_ reaction almost exclusively forms chain-terminating products, whereas the 2-methylpropan-1-yl + O_2_ reaction forms mostly chain-propagating and/or -braching products, depending on the conditions.

## Supplementary Material









## Notes

Reduced model (Int1 ⇔ Int3) equilibrium constant as well as Chebyshev and PLOG parametrizations for *i*-C_4_H_9_ + O_2_ reaction (TXT)
Tables detailing the conditions and result of each measurement, as well as figures relevant to the main text (PDF)
Full and reduced Master-equation (MESMER) input files for *i*-C_4_H_9_ + O_2_ and *t*-C_4_H_9_ + O_2_ reactions (ZIP)
Reduced model Chebyshev and PLOG parametrizations for *t*-C_4_H_9_ + O_2_ reaction (TXT)

## References

[1] Zádor J., Taatjes C. A., Fernandes R. X. (2011). Kinetics
of elementary reactions
in low-temperature autoignition chemistry. Prog.
Energy Combustion Sci..

[2] Atri G. M., Baldwin R. R., Evans G. A., Walker R. W. (1978). Decomposition
of
2,2,3,3-tetramethylbutane in the presence of oxygen. J. Chem. Soc., Faraday Trans. 1.

[3] Xing L., Wang X., Cheng Z. (2020). Kinetics of
iso-butyl radical reaction
with O_2_ in combustion: The first and second oxygen addition. Combust. Flame.

[4] Wu D., Bayes K. D. (1986). Rate constants for
the reactions of isobutyl, neopentyl,
cyclopentyl, and cyclohexyl radicals with molecular oxygen. Int. J. Chem. Kinet..

[5] Eskola A. J., Pekkanen T. T., Lendvay G., Timonen R. S. (2023). An experimental
and theoretical kinetic study of t-butyl radical reaction with molecular
oxygen. Proc. Combust. Inst..

[6] Eskola A. J., Pekkanen T. T., Joshi S. P., Timonen R. S., Klippenstein S. J. (2019). Kinetics
of 1-butyl and 2-butyl radical reactions with molecular oxygen: Experiment
and theory. Proc. Combust. Inst..

[7] Lenhardt T. M., McDade C. E., Bayes K. D. (1980). Rates of
reaction of butyl radicals
with molecular oxygen. J. Chem. Phys..

[8] Dilger H., Stolmár M., Tregenna-Piggott P. L., Roduner E., Reid I. D. (1997). Gas phase
addition kinetics of the tert-butyl radical to oxygen. Ber. Bunsenges. Phys. Chem..

[9] Baker R. R., Baldwin R. R., Walker R. W. (1978). Addition
of i-butane to slowly reacting
mixtures of hydrogen and oxygen at 480 °C. J. Chem. Soc., Faraday Trans. 1.

[10] Evans G. A., Walker R. W. (1979). Reaction of t-butyl
radicals with hydrogen and with
oxygen. J. Chem. Soc., Faraday Trans. 1.

[11] Slagle I. R., Ratajczak E., Gutman D. (1986). Study of the thermochemistry of the
ethyl + molecular oxygen ⇌ ethylperoxy (C_2_H_5_O_2_) and tert-butyl + molecular oxygen ⇌
tert-butylperoxy (tert-C_4_H_9_O_2_) reactions
and of the trend in the alkylperoxy bond strengths. J. Phys. Chem. A.

[12] DeSain J. D., Taatjes C. A., Miller J. A., Klippenstein S. J., Hahn D. K. (2001). Infrared frequency-modulation probing of product formation
in alkyl + O_2_ reactions. Part IV. Reactions of propyl and
butyl radicals with O_2_. Faraday Discuss..

[13] Miyoshi A. (2012). Molecular
size dependent falloff rate constants for the recombination reactions
of alkyl radicals with O2 and implications for simplified kinetics
of alkylperoxy radicals. Int. J. Chem. Kinet..

[14] Moore K. B., Kevin B., Turney J. M., Schaefer I., Henry F. (2017). The fate of
the tert-butyl radical in low-temperature autoignition reactions. J. Chem. Phys..

[15] Eskola A. J., Timonen R. S. (2003). Kinetics of the
reactions of vinyl radicals with molecular
oxygen and chlorine at temperatures 200–362 K. Phys. Chem. Chem. Phys..

[16] Pekkanen T. T., Ramu E. A., Timonen R. S., Eskola A. J., Lendvay G. (2024). Addition and
elimination reactions on the C_4_H_9_
^•^ potential-energy surface: Experiments and master-equation analysis
of literature data. Proc. Combust. Inst..

[17] Yu H. S., He X., Louis Li S., G Truhlar D. (2016). MN15: A Kohn-Sham Global-Hybrid Exchange-Correlation
Density Functional with Broad Accuracy for Multi-Reference and Single-Reference
Systems and Noncovalent Interactions. Chem.
Sci..

[18] Weigend F., Ahlrichs R. (2005). Balanced basis sets
of split valence, triple zeta valence
and quadruple zeta valence quality for H to Rn: Design and assessment
of accuracy. Phys. Chem. Chem. Phys..

[19] Watts J. D., Gauss J., Bartlett R. J. (1993). Coupled-cluster
methods with noniterative
triple excitations for restricted open-shell Hartree-Fock and other
general single determinant reference functions. Energies and analytical
gradients. J. Chem. Phys..

[20] Dunning T. H. (1989). Gaussian
basis sets for use in correlated molecular calculations. I. The atoms
boron through neon and hydrogen. J. Chem. Phys..

[21] Halkier A., Helgaker T., Jørgensen P., Klopper W., Olsen J. (1999). Basis-set
convergence of the energy in molecular Hartree-Fock calculations. Chem. Phys. Lett..

[22] Helgaker T., Klopper W., Koch H., Noga J. (1997). Basis-set
convergence
of correlated calculations on water. J. Chem.
Phys..

[23] Frisch, M. J. ; Trucks, G. W. ; Schlegel, H. B. ; Scuseria, G. E. ; Robb, M. A. ; Cheeseman, J. R. ; Scalmani, G. ; Barone, V. ; Petersson, G. A. ; Nakatsuji, H. ; Li, X. ; Caricato, M. ; Marenich, A. V. ; Bloino, J. ; Janesko, B. G. ; Gomperts, R. ; Mennucci, B. ; Hratchian, H. P. ; Ortiz, J. V. ; Izmaylov, A. F. ; Sonnenberg, J. L. ; Williams-Young, D. ; Ding, F. ; Lipparini, F. ; Egidi, F. ; Goings, J. ; Peng, B. ; Petrone, A. ; Henderson, T. ; Ranasinghe, D. ; Zakrzewski, V. G. ; Gao, J. ; Rega, N. ; Zheng, G. ; Liang, W. ; Hada, M. ; Ehara, M. ; Toyota, K. ; Fukuda, R. ; Hasegawa, J. ; Ishida, M. ; Nakajima, T. ; Honda, Y. ; Kitao, O. ; Nakai, H. ; Vreven, T. ; Throssell, K. ; Montgomery, J. A., Jr. ; Peralta, J. E. ; Ogliaro, F. ; Bearpark, M. J. ; Heyd, J. J. ; Brothers, E. N. ; Kudin, K. N. ; Staroverov, V. N. ; Keith, T. A. ; Kobayashi, R. ; Normand, J. ; Raghavachari, K. ; Rendell, A. P. ; Burant, J. C. ; Iyengar, S. S. ; Tomasi, J. ; Cossi, M. ; Millam, J. M. ; Klene, M. ; Adamo, C. ; Cammi, R. ; Ochterski, J. W. ; Martin, R. L. ; Morokuma, K. ; Farkas, O. ; Foresman, J. B. ; Fox, D. J. Gaussian Inc 16 Revision C.; Gaussian Inc.: Wallingford CT, 2016.

[24] Kállay M., Nagy P. R., Mester D., Rolik Z., Samu G., Csontos J., Csóka J., Szabó P. B., Gyevi-Nagy L., Hégely B., Ladjánszki I., Szegedy L., Ladóczki B., Petrov K., Farkas M., Mezei P. D., Ganyecz A. (2020). The MRCC program system: Accurate
quantum chemistry from water to proteins. J.
Chem. Phys..

[25] Kállay, M. ; Nagy, P. R. ; Mester, D. ; Gyevi-Nagy, L. ; Csóka, J. ; Szabó, P. B. ; Rolik, Z. ; Samu, G. ; Hégely, B. ; Ladóczki, B. ; Petrov, K. ; Csontos, J. ; Ganyecz, A. ; Ladjánszki, I. ; Szegedy, L. ; Farkas, M. ; Mezei, P. D. ; Horváth, R. A. ; Lőrincz, B. D. MRCC, a quantum chemical program suite. See https://mrcc.hu.

[26] Kállay M., Gauss J. (2008). Approximate treatment
of higher excitations in coupled-cluster theory.
II. Extension to general single-determinant reference functions and
improved approaches for the canonical Hartree-Fock case. J. Chem. Phys..

[27] Glowacki D. R., Liang C.-H., Morley C., Pilling M. J., Robertson S. H. (2012). MESMER:
An Open-Source Master Equation Solver for Multi-Energy Well Reactions. J. Phys. Chem. A.

[28] Davies J. W., Green N. J., Pilling M. J. (1986). The testing of models for unimolecular
decomposition via inverse Laplace transformation of experimental recombination
rate data. Chem. Phys. Lett..

[29] Forst W. (1972). Unimolecular
rate theory test in thermal reactions. J. Phys.
Chem. A.

[30] Poling, B. E. ; Prausnitz, J. M. ; O’Connell, J. P. The Properties of Gases and Liquids, 5 ^th^ ed.; McGraw-Hill: New York, 2001.

[31] Gao C. W., Allen J. W., Green W. H., West R. H. (2016). Reaction Mechanism
Generator: Automatic construction of chemical kinetic mechanisms. Comput. Phys. Commun..

[32] Bao, J. L. ; Zheng, J. ; Alecu, I. M. ; Lynch, B. J. ; Zhao, Y. ; Truhlar, D. G. Database of Frequency Scale Factors for Electronic Model Chemistries. https://comp.chem.umn.edu/freqscale/version3b2.htm, Version 5, Accessed: September 9, 2026.

[33] Gang J., Pilling M. J., Robertson S. H. (1998). Monte Carlo calculation of partition
functions for straight chain alkanes. Chem.
Phys..

[34] Bartis J. T., Widom B. (1974). Stochastic models of the interconversion of three or more chemical
species. J. Chem. Phys..

[35] Robertson S. H., Pilling M. J., Jitariu L. C., Hillier I. H. (2007). Master equation
methods for multiple well systems: application to the 1-,2-pentyl
system. Phys. Chem. Chem. Phys..

[36] Medeiros D. J., Robertson S. H., Blitz M. A., Seakins P. W. (2020). Direct Trace Fitting
of Experimental Data Using the Master Equation: Testing Theory and
Experiments on the OH + C_2_H_4_ Reaction. J. Phys. Chem. A.

[37] Knyazev V. D., Slagle I. R. (1998). Thermochemistry of the R-O_2_ Bond in Alkyl
and Chloroalkyl Peroxy Radicals. J. Phys. Chem.
A.

[38] Miller J.
A., Klippenstein S. J., Robertson S. H., Pilling M. J., Shannon R., Zádor J., Jasper A. W., Goldsmith C. F., Burke M. P. (2016). Comment on “When
Rate Constants Are Not Enough”. J. Phys.
Chem. A.

[39] Hansen A. S., Bhagde T., Moore K. B., Moberg D. R., Jasper A. W., Georgievskii Y., Vansco M. F., Klippenstein S. J., Lester M. I. (2021). Watching a hydroperoxyalkyl radical (^•^QOOH) dissociate. Science.

[40] Pekkanen T. T., Timonen R. S., Ramu E. A., Lendvay G., Eskola A. J. (2023). Temperature
and Pressure Dependence of the Reaction between Ethyl Radical and
Molecular Oxygen: Experiments and Master Equation Simulations. J. Phys. Chem. A.

[41] Miller J. A., Klippenstein S. J. (2001). The reaction between ethyl and molecular
oxygen II:
Further analysis. Int. J. Chem. Kinet..

[42] Fang Q., Zhang Y., Xia J., Li Y. (2022). Theoretical Investigation
on H-Abstraction Reactions of Silanes with H and CH_3_ Attacking:
A Comparative Study with Alkane Counterparts. ACS Omega.

[43] Klippenstein S. J. (2017). From theoretical
reaction dynamics to chemical modeling of combustion. Proc. Combust. Inst..

[44] Eskola A. J., Pekkanen T. T., Salomaa P. S., Lendvay G., Timonen R. S. (2024). Revising
the kinetics of the *n*-C_3_H_7_ +
O_2_ reaction: A combined experimental and computational
study. Proc. Combust. Inst..

[45] Eskola A. J., Ruotsalainen J. S., Pekkanen T. T., Zalte A. S., Rissanen M. P., Green W. H. (2026). Kinetics
of the reaction between propan-2-yl radical
and molecular oxygen: experiments and master-equation simulations. Proc. Combust. Inst..

[46] Fernandes R. X., Luther K., Troe J. (2006). Falloff Curves
for the Reaction in
the Pressure Range 2–1000 bar and the Temperature Range 300–700K. J. Phys. Chem. A.

