# Supplementary material for: An Experimental and Computational Investigation of the Kinetics of Branched Butyl Radicals 2‑Methylpropan-1-yl and 2‑Methylpropan-2-yl with Molecular Oxygen
Source: J Phys Chem A. 2026 Oct 6;130(42):8348–60. doi: 10.1021/acs.jpca.6c05216 (PMC13700103; doi:10.1021/acs.jpca.6c05216)
Supplement: Supplementary file 1 [file jp6c05216_si_002.pdf]

An Experimental and Computational Investigation of the Kinetics of Branched Butyl Radicals 2-Methylpropan-1-yl and 2-Methylpropan-2-yl with Molecular Oxygen

Timo T. Pekkanen[a,b,c], Raimo S. Timonen[a], György Lendvay[c], William H. Green[c], Arkke J. Eskola[a,*]

[a]*Department of Chemistry, University of Helsinki, P.O. Box 55 (A.I. Virtasen aukio 1), 00014 Helsinki, Finland*
[b]*Institute of Materials and Environmental Chemistry, Research Centre for Natural Sciences, Magyar Tudósok krt. 2., Budapest H-1117, Hungary*
[c]*Department of Chemical Engineering, Massachusetts Institute of Technology, Cambridge, MA 02139, United States of America*

Supplemental Material

---

*Corresponding author:
Email address: `arkke.eskola@helsinki.fi` (Arkke J. Eskola)

Table S1: The experimental conditions and results of i-$C_4H_9^\bullet$ + O_2 bimolecular-rate-coefficient measurements.

T (K)	p (Torr)	[M] (cm^{-3}) 10^{16}	[Prec] (cm^{-3}) 10^{11}	[O_2] (cm^{-3}) 10^{12}	k'^{a} (s^{-1})	$k_w{}^{b}$ (s^{-1})	$k_w{}^{c}$ (s^{-1})	k^{d} ($cm^3\ s^{-1}$) 10^{-13}
220[e,j,k,l]	0.40	1.76	150	7.88 − 21.6	97.0 − 186	45.5 ± 4.1	45.3 ± 1.3	65.0 ± 1.0
223[e,i,k,l]	0.42	1.80	2.4	5.04 − 19.7	37.6 − 145	16.1 ± 1.6	10.7 ± 7.9	61.1 ± 7.6
220[e,j,k,l]	0.85	3.74	130	6.59 − 22.2	99.3 − 196	43.8 ± 3.5	46.7 ± 3.0	68.3 ± 2.5
223[e,i,k,l]	0.86	3.72	3.7	9.44 − 26.7	95.4 − 216	25.1 ± 1.5	21.5 ± 9.4	70.0 ± 5.9
220[e,j,k,l]	1.44	6.30	130	5.49 − 22.0	86.5 − 214	43.6 ± 3.1	40.9 ± 5.5	73.4 ± 4.4
223[e,i,k,l]	1.47	6.37	4.3	5.33 − 17.0	55.7 − 119	20.5 ± 1.0	22.2 ± 2.0	59.1 ± 2.1
220[f,j,k,l]	2.80	12.3	190	4.06 − 16.3	47.7 − 126	22.1 ± 1.9	23.3 ± 5.1	67.7 ± 5.8
220[f,j,k,l]	5.67	24.9	190	3.99 − 16.6	44.1 − 133	17.5 ± 1.8	18.4 ± 4.4	68.6 ± 4.7
220[f,j,k,l]	11.6	50.9	110	3.48 − 18.3	43.7 − 145	16.6 ± 1.4	18.8 ± 2.3	68.7 ± 2.3
243[e,i,k,l]	0.46	1.81	3.5	15.8 − 33.8	99.2 − 207	17.2 ± 1.7	17.3 ± 6.7	55.1 ± 3.3
243[e,i,k,l]	0.94	3.75	3.6	12.5 − 31.2	87.9 − 198	19.4 ± 1.4	19.5 ± 1.4	56.0 ± 0.7
243[e,i,k,l]	1.58	6.26	4.0	11.1 − 27.3	95.5 − 179	20.8 ± 1.1	27.2 ± 6.9	57.4 ± 3.9
255[e,j,k,l]	0.50	1.87	160	8.05 − 33.3	79.3 − 200	43.9 ± 2.9	44.2 ± 2.2	48.0 ± 1.3
255[e,j,k,l]	1.00	3.80	170	7.99 − 22.7	71.4 − 140	36.8 ± 2.4	37.9 ± 3.3	46.8 ± 2.5
255[f,j,k,l]	1.58	5.99	140	5.79 − 21.4	44.1 − 124	14.6 ± 2.0	14.3 ± 3.0	48.3 ± 2.5
255[e,j,k,l]	1.67	6.34	85	7.78 − 25.5	72.7 − 163	32.8 ± 2.4	35.8 ± 5.2	49.9 ± 3.6
255[f,j,k,l]	3.25	12.3	140	4.03 − 16.9	35.4 − 100	15.8 ± 1.6	15.4 ± 1.3	51.1 ± 1.4
255[f,j,k,l]	6.68	25.3	180	4.56 − 17.9	35.6 − 110	15.7 ± 1.8	15.7 ± 3.1	54.0 ± 3.0
280[e,i,k,l]	1.09	3.76	3.8	11.7 − 24.9	62.0 − 128	14.2 ± 0.9	11.7 ± 5.1	45.7 ± 3.1
304[g,j,k,l]	0.37	1.17	50	6.72 − 31.8	31.6 − 106	12.6 ± 1.3	12.9 ± 1.5	30.2 ± 0.8
298[e,j,k,l]	0.57	1.86	67	9.51 − 32.6	41.0 − 116	8.42 ± 0.97	8.96 ± 0.82	33.3 ± 0.4
298[e,i,k,l]	0.57	1.86	2.0	17.8 − 43.1	84.5 − 155	14.6 ± 1.6	18.1 ± 4.3	33.7 ± 1.7
304[g,j,k,l]	0.77	2.44	50	6.88 − 30.4	32.6 − 103	11.9 ± 1.3	12.3 ± 2.0	31.1 ± 1.2
302[e,i,k,l]	1.16	3.70	16	3.99 − 31.6	18.3 − 145	7.91 ± 0.81	5.34 ± 3.30	42.0 ± 1.9
298[e,j,k,l]	1.16	3.76	65	7.47 − 37.8	32.8 − 144	5.57 ± 0.66	5.97 ± 1.31	37.2 ± 6.5
304[g,j,k,l]	1.19	3.77	40	6.19 − 32.7	38.2 − 127	14.2 ± 0.5	16.1 ± 1.6	33.9 ± 0.9
305[h,i,k,l]	1.89	5.98	20	5.10 − 32.0	35.9 − 141	20.1 ± 0.8	19.4 ± 3.1	38.3 ± 1.9
298[f,j,k,l]	1.91	6.20	140	7.25 − 27.9	45.5 − 123	18.2 ± 2.0	18.4 ± 4.0	39.6 ± 2.4
298[e,i,k,l]	1.97	6.37	1.2	16.2 − 35.0	70.3 − 146	9.30 ± 1.21	9.34 ± 1.54	39.6 ± 0.8
298[e,j,k,l]	1.97	6.39	46	6.77 − 32.0	28.2 − 124	5.62 ± 0.94	6.49 ± 0.37	38.6 ± 2.1
304[g,j,k,l]	1.98	6.28	45	7.02 − 31.7	34.4 − 121	13.3 ± 1.2	13.6 ± 2.9	35.4 ± 1.6
298[f,i,k,l]	1.98	6.42	9.4	4.76 − 41.8	55.1 − 207	27.2 ± 0.8	31.9 ± 2.7	42.5 ± 1.3
310[h,j,k,l]	2.97	9.24	31	6.04 − 26.7	40.9 − 107	19.5 ± 1.3	21.6 ± 3.4	34.2 ± 2.2
298[f,i,k,l]	3.28	10.4	3.6	12.8 − 32.7	75.0 − 154	16.0 ± 0.9	17.5 ± 4.8	41.9 ± 2.2
298[f,j,k,l]	3.78	12.3	64	10.1 − 27.2	43.4 − 121	9.96 ± 1.18	7.49 ± 3.36	39.9 ± 2.1
298[f,i,k,l]	4.54	14.7	11	27.1 − 90.1	151 − 428	26.9 ± 1.0	26.2 ± 5.0	44.5 ± 0.9
298[f,i,k,l]	4.55	14.8	5.2	8.03 − 37.2	60.7 − 191	24.8 ± 1.2	27.1 ± 3.6	45.3 ± 1.9
298[f,i,k,l]	4.59	14.9	7.2	9.04 − 42.6	67.5 − 221	22.8 ± 0.9	22.5 ± 2.3	45.9 ± 0.9
298[f,j,k,l]	7.89	25.6	120	6.75 − 20.9	44.6 − 119	18.5 ± 1.2	17.2 ± 1.8	47.6 ± 1.5
355[e,j,k,l]	0.68	1.86	100	18.4 − 42.2	68.6 − 121	17.6 ± 1.5	19.6 ± 4.3	26.8 ± 1.7
355[e,j,k,l]	1.41	3.84	65	8.20 − 38.6	33.9 − 126	13.5 ± 1.7	12.3 ± 1.9	28.4 ± 0.9
355[f,j,k,l]	2.26	6.15	110	7.57 − 35.2	39.0 − 121	17.9 ± 1.4	18.1 ± 1.2	29.4 ± 0.6
355[e,j,k,l]	2.37	6.49	58	7.56 − 34.3	32.1 − 112	10.6 ± 1.5	11.0 ± 1.3	29.7 ± 0.6

355[f,j,k,l]	4.63	12.6	76	$7.44 - 34.1$	$36.8 - 124$	14.6 ± 1.3	16.3 ± 4.1	31.7 ± 2.2
355[f,j,k,l]	4.63	12.6	110	$8.04 - 34.9$	$46.1 - 133$	15.3 ± 1.3	17.8 ± 5.3	31.9 ± 2.5
355[f,j,k,l]	9.40	25.5	65	$8.07 - 32.8$	$42.1 - 124$	14.4 ± 1.2	15.0 ± 1.2	33.2 ± 0.6
363[e,i,k,l]	0.70	1.87	2.6	$16.4 - 33.7$	$57.6 - 97.6$	8.92 ± 1.32	11.4 ± 5.2	25.6 ± 2.3
363[f,i,k,l]	4.14	11.0	–	$30.3 - 63.2$	$139 - 203$	23.4 ± 1.2	25.1 ± 2.5	28.7 ± 0.6
363[f,i,k,l]	5.58	14.8	13	$12.4 - 52.7$	$73.6 - 211$	25.1 ± 0.8	28.9 ± 5.3	34.0 ± 1.8
420[g,j,k,l]	0.77	1.77	36	$19.5 - 58.7$	$46.2 - 112$	8.16 ± 1.39	8.62 ± 1.6	17.6 ± 0.4
420[g,j,k,l]	1.48	3.39	41	$14.1 - 49.1$	$34.6 - 119$	6.85 ± 1.27	8.56 ± 5.2	20.3 ± 1.5
416[g,j,k,l]	2.37	5.51	32	$12.3 - 52.0$	$32.8 - 122$	7.43 ± 1.38	6.73 ± 0.86	22.2 ± 0.3
420[h,j,k,l]	2.39	5.49	66	$11.4 - 45.5$	$41.4 - 118$	14.8 ± 1.5	16.3 ± 2.6	23.0 ± 1.0
420[h,j,k,l]	4.88	11.2	110	$11.5 - 43.1$	$45.9 - 137$	15.7 ± 1.6	16.7 ± 3.0	27.7 ± 1.3
498[g,j,k,l]	0.91	1.76	35	$13.9 - 81.0$	$26.4 - 85.3$	8.38 ± 1.74	11.0 ± 4.9	10.4 ± 1.2
495[g,j,k,l]	1.72	3.35	43	$19.4 - 79.8$	$32.9 - 108$	6.44 ± 1.44	8.23 ± 6.69	12.6 ± 1.5
493[h,j,k,l]	1.93	3.78	67	$17.6 - 54.2$	$31.4 - 98.7$	11.4 ± 1.4	9.55 ± 2.95	16.5 ± 0.9
489[h,j,k,l]	2.49	4.91	100	$13.9 - 59.7$	$41.3 - 107$	14.6 ± 1.3	17.5 ± 4.9	13.5 ± 1.4
492[g,j,k,l]	2.80	5.49	36	$15.0 - 76.0$	$30.1 - 128$	10.7 ± 1.3	8.43 ± 2.92	15.1 ± 0.7
493[h,j,k,l]	3.05	5.97	63	$5.43 - 60.7$	$20.9 - 113$	9.42 ± 1.18	8.86 ± 1.58	17.3 ± 0.5
489[h,j,k,l]	4.85	9.58	84	$14.0 - 44.1$	$48.9 - 109$	15.5 ± 1.3	14.4 ± 3.3	20.2 ± 1.2
493[h,j,k,l]	4.95	9.68	35	$16.0 - 58.8$	$33.5 - 110$	10.6 ± 1.5	10.4 ± 3.1	18.6 ± 1.0
550[g,j,k,l]	1.01	1.77	42	$19.1 - 108$		14.3 ± 1.9		5 Traces
553[g,j,k,l]	1.93	3.36	43	$19.4 - 99.1$		15.8 ± 1.7		5 Traces
546[h,j,k,l]	1.96	3.47	55	$20.2 - 84.0$		19.6 ± 1.8		6 Traces
555[h,j,k,l]	3.97	6.24	62	$17.0 - 76.9$		19.4 ± 1.4		5 Traces
546[h,j,k,l]	5.89	10.6	74	$22.5 - 68.4$		17.6 ± 1.5		6 Traces
304[g,j,k,m]	0.48	1.54	47	$6.87 - 32.3$	$44.7 - 135$	15.7 ± 1.5	17.8 ± 3.9	35.4 ± 2.1
298[e,i,k,m]	0.58	1.87	25	$9.98 - 33.9$	$60.6 - 149$	21.6 ± 0.8	22.0 ± 1.3	37.7 ± 0.6
304[g,j,k,m]	0.97	3.14	70	$6.25 - 23.7$	$37.8 - 123$	13.5 ± 1.7	12.3 ± 3.3	40.4 ± 2.5
298[e,i,k,m]	1.18	3.81	27	$6.31 - 25.2$	$46.0 - 124$	21.8 ± 1.0	21.2 ± 1.2	40.9 ± 0.8
304[g,j,k,m]	1.48	4.70	68	$5.71 - 25.2$	$44.6 - 122$	19.8 ± 1.9	20.0 ± 0.9	40.8 ± 0.7
302[h,j,k,m]	2.32	7.42	92	$9.90 - 36.8$	$62.1 - 176$	20.4 ± 2.5	19.4 ± 2.5	42.4 ± 1.3
494[g,i,k,m]	0.96	1.88	33	$27.4 - 84.7$	$86.1 - 178$	44.2 ± 3.7	45.1 ± 2.3	16.0 ± 0.5
494[g,i,k,m]	1.99	3.88	44	$30.8 - 87.3$	$129 - 238$	54.6 ± 4.1	55.2 ± 4.2	21.0 ± 0.8
498[h,i,k,m]	2.89	5.60	83	$12.3 - 41.1$	$110 - 218$	74.9 ± 6.0	72.9 ± 3.1	33.3 ± 1.4

[a] The pseudo first-order rate coefficient $k' = k[O_2] + k_w$.
[b] Average of measured wall rates. Stated uncertainty is the average standard error (1σ) of the fits. Wall rate is the radical decay rate in the absence of added oxygen.
[c] Wall rate determined from the linear fit y-axis intercept of the bimolecular plot. Stated uncertainty is the standard error (1σ) of the fit.
[d] Experimentally determined bimolecular rate coefficient (slope of the bimolecular plot). Stated uncertainty is the standard error (1σ) of the linear fit. Estimated overall uncertainty is ±20 %.
[e] Reactor: $d = 1.70$ cm, stainless steel with halocarbon.wax coating.
[f] Reactor: $d = 0.80$ cm, stainless steel with halocarbon.wax coating.
[g] Reactor: $d = 1.70$ cm, quartz with boric.oxide coating.
[h] Reactor: $d = 0.85$ cm, quartz with boric.oxide coating.
[i] Radical precursor is 1-iodo-2-methylpropane kept at around $T_{Pr} = -6$ °C. 248 nm photolysis.
[j] Radical precursor is 4-methylpentan-2-one kept at around $T_{Pr} = -6$ °C, 193 nm photolysis.
[k] Detection: Cl/BaF_2.
[l] He bath gas.
[m] N_2 bath gas.

Table S2: The experimental conditions and results of t-$C_4H_9^{\bullet}$ + O_2 bimolecular-rate-coefficient measurements.

T (K)	p (Torr)	[M] (cm^{-3}) 10^{16}	[Prec] (cm^{-3}) 10^{11}	[O_2] (cm^{-3}) 10^{12}	k'[a] (s^{-1})	k_w[b] (s^{-1})	k_w[c] (s^{-1})	k[d] ($cm^3\ s^{-1}$) 10^{-13}
415[g,j,l,n]	0.98	2.28	81	3.86 – 16.5	36.4 – 123	10.1 ± 0.5	10.2 ± 3.0	65.6 ± 3.3
415[g,j,l,n]	1.97	4.58	76	6.06 – 15.8	50.3 – 119	8.28 ± 0.47	8.42 ± 0.81	70.9 ± 0.9
417[h,j,l,n]	4.06	9.40	70	10.0 – 32.1	102 – 269	20.3 ± 0.8	21.9 ± 2.2	77.3 ± 1.2
417[h,j,l,n]	8.08	18.7	70	9.66 – 28.2	127 – 285	20.7 ± 0.5	24.9 ± 4.9	96.0 ± 2.9
497[g,j,l,n]	1.23	2.38	64	7.27 – 36.3	35.9 – 134	10.2 ± 0.4	11.5 ± 1.9	33.5 ± 0.9
495[g,j,l,n]	2.48	4.84	61	7.24 – 36.9	41.9 – 154	8.30 ± 0.38	10.6 ± 1.9	39.9 ± 0.9
495[h,j,l,n]	4.97	9.70	44	14.2 – 34.9	90.8 – 188	17.5 ± 0.7	19.3 ± 2.6	48.9 ± 1.2
495[h,j,l,n]	9.93	19.4	51	19.7 – 43.4	135 – 263	18.9 ± 1.1	20.2 ± 2.4	57.1 ± 0.9
650[g,j,l,n]	1.22	1.81	63	26.8 – 93.5	48.9 – 109	13.7 ± 0.6	16.1 ± 2.1	9.92 ± 0.37
650[g,j,l,n]	2.43	3.62	59	26.3 – 133	39.6 – 130	14.1 ± 0.6	17.5 ± 4.2	9.24 ± 0.59
646[h,j,l,n]	4.80	7.18	55	51.0 – 168	79.5 – 176	24.4 ± 0.9	27.4 ± 3.0	9.19 ± 0.29
647[h,j,l,n]	9.81	14.6	65	80.0 – 209	106 – 209	30.1 ± 1.6	31.6 ± 3.1	9.00 ± 0.29
304[i,k,m,o]	0.52	1.66	1600	2.44 – 7.88	72.0 – 150	27.5 ± 2.3	28.5 ± 2.1	157 ± 5
298[e,k,m,o]	0.57	1.86	97	1.94 – 7.75	44.1 – 125	14.9 ± 1.1	15.4 ± 2.0	143 ± 5
304[i,k,m,o]	0.78	2.48	1100	2.34 – 8.13	57.6 – 149	21.1 ± 1.3	21.8 ± 1.5	157 ± 3
304[i,k,m,o]	1.17	3.72	1200	2.29 – 7.24	68.6 – 143	25.2 ± 1.6	24.5 ± 3.1	155 ± 7
298[e,j,m,o]	1.17	3.79	141	1.79 – 6.99	48.8 – 141	16.9 ± 1.1	17.0 ± 0.9	180 ± 2
298[f,j,m,o]	1.68	5.48	161	4.02 – 14.8	101 – 280	26.1 ± 2.0	28.5 ± 4.0	176 ± 5
298[f,j,m,o]	3.52	11.4	182	4.05 – 12.1	107 – 251	29.9 ± 2.0	32.6 ± 5.2	186 ± 7
498[g,j,l,o]	0.97	1.88	88	7.24 – 40.0	31.9 – 123	5.57 ± 0.62	8.37 ± 2.21	29.8 ± 1.0
498[g,j,l,o]	1.97	3.82	81	5.40 – 37.7	27.0 – 142	5.90 ± 0.63	7.91 ± 2.43	37.1 ± 1.2
498[h,j,l,o]	2.88	5.78	32	13.8 – 40.8	81.0 – 194	22.9 ± 1.6	22.9 ± 2.8	40.9 ± 1.2

[a] The pseudo first-order rate coefficient $k' = k[O_2] + k_w$.

[b] Average of measured wall rates. Stated uncertainty is the average standard error (1σ) of the fits. Wall rate is the radical decay rate in the absence of added oxygen.

[c] Wall rate determined from the linear fit y-axis intercept of the bimolecular plot. Stated uncertainty is the standard error (1σ) of the fit.

[d] Experimentally determined bimolecular rate coefficient (slope of the bimolecular plot). Stated uncertainty is the standard error (1σ) of the linear fit. Estimated overall uncertainty is ±20 %.

[e] Reactor: d = 1.70 cm, stainless steel with halocarbon-wax coating.

[f] Reactor: d = 1.80 cm, stainless steel, halocarbon-wax coating.

[g] Reactor: d = 1.70 cm, quartz, boric-oxide coating.

[h] Reactor: d = 0.85 cm, quartz, boric-oxide coating.

[i] Reactor: d = 1.70 cm, quartz, polydimethylsiloxane coating.

[j] Precursor: 3,3-dimethylbutan-2-one (pinacolone) kept at around −7 °C. ArF (λ = 193 nm) used for photolysis.

[k] Precursor: 2-bromo-2-methylpropane kept at around −11 °C. KrF (λ = 248 nm) used for photolysis.

[l] Detection: chlorine lamp with a BaF_2 window.

[m] Detection: nitrogen lamp with a quartz window.

[n] Bath gas: helium.

[o] Bath gas: nitrogen.

Table S3: The experimental conditions and results of t-$C_4H_9^\bullet$ + O_2 measurements in the intermediate temperature regime. The traces are multi-exponential, and the fitting parameters reported here are for the double-exponential function $[t\text{-}C_4H_9^\bullet] = A\mathrm{e}^{-\lambda_1 t} + B\mathrm{e}^{-\lambda_2 t}$.

T (K)	p (Torr)	[M] (cm^3) 10^{16}	[Prec] (cm^3) 10^{11}	[O_2] (cm^3) 10^{12}	k_w[a] (s^{-1})	λ_1 (s^{-1})	λ_2 (s^{-1})	A	B
548[b,c,d,e]	5.89	10.4	85	68.8	18.2 ± 0.8	233 ± 16	42.1 ± 9.4	55.7 ± 1.9	7.09 ± 2.06
558[b,c,d,e]	5.88	10.2	85	68.0	18.2 ± 0.8	283 ± 55	89.2 ± 13.7	50.8 ± 8.6	31.5 ± 9.8
568[b,c,d,e]	5.89	10.0	85	67.2	18.2 ± 0.8	317 ± 46	88.9 ± 8.5	50.0 ± 5.4	35.3 ± 6.2
570[b,c,d,e]	5.91	10.0	59	149	23.0 ± 1.0	349 ± 25	59.1 ± 14.8	23.7 ± 0.9	2.60 ± 0.85
580[b,c,d,e]	5.92	9.85	85	66.5	18.2 ± 0.8	337 ± 70	82.1 ± 7.1	38.7 ± 4.7	38.0 ± 5.5

[a] Average of measured wall rates. Stated uncertainty is the average standard error (1σ) of the fits. Wall rate is the radical decay rate in the absence of added oxygen.
[b] Reactor: $d = 0.85$ cm, quartz, boric-oxide coating.
[c] Precursor: 3,3-dimethylbutan-2-one (pinacolone) kept at around -7 °C. ArF ($\lambda = 193$ nm) used for photolysis.
[d] Detection: chlorine lamp with a BaF_2 window.
[e] Bath gas: helium.

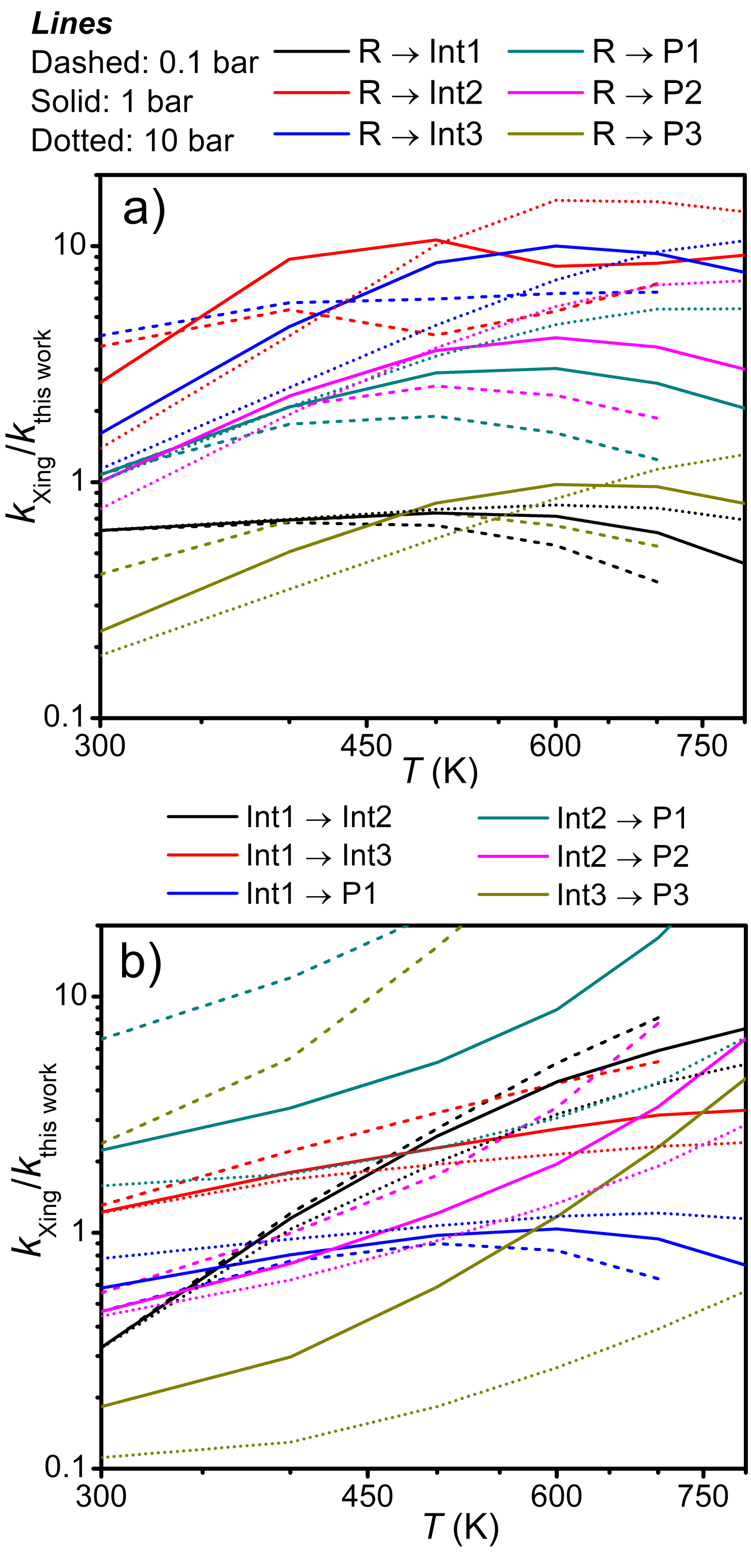

Figure S1: Comparison between current and Xing et al.[1] results for **a)** bimolecular and **b)** unimolecular rate coefficients. The results are presented as the ratio of their result to ours. We ran our simulations in N_2, whereas they utilised Ar.

[1]Lili Xing, Xuetao Wang, and Zhanjun Cheng, *Kinetics of iso-butyl radical reaction with O_2 in combustion: The first and second oxygen addition*, Combust. Flame., 220:429-438, 2020, `https://doi.org/10.1016/j.combustflame.2020.07.014`

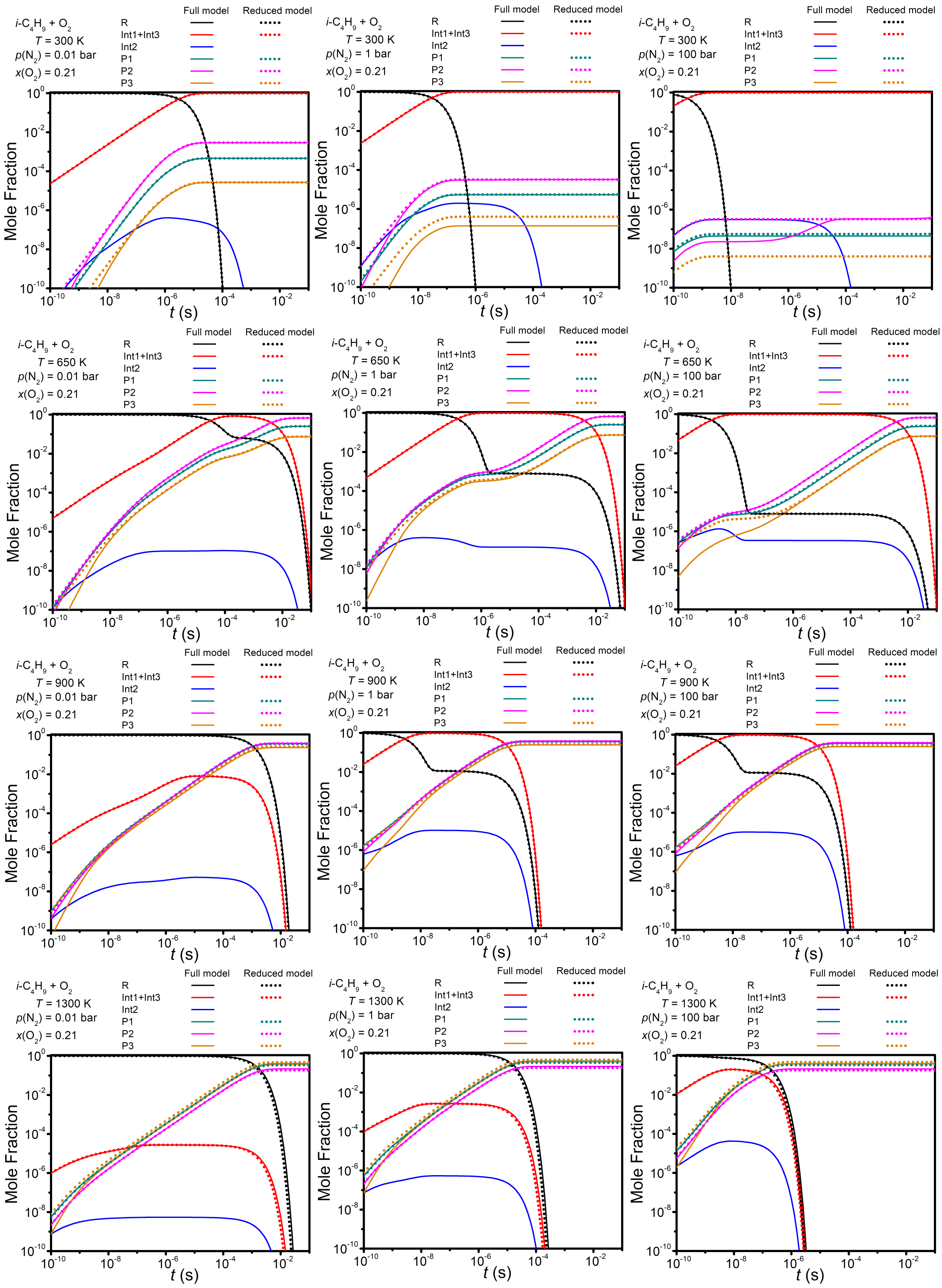

Figure S2: Concentration-time profiles simulated with the full (solid lines) and reduced (dotted lines) i-$C_4H_9^{\bullet}$ + O_2 models at different p_{N_2}, and T. Note that the profiles output by the two models nearly perfectly overlap.

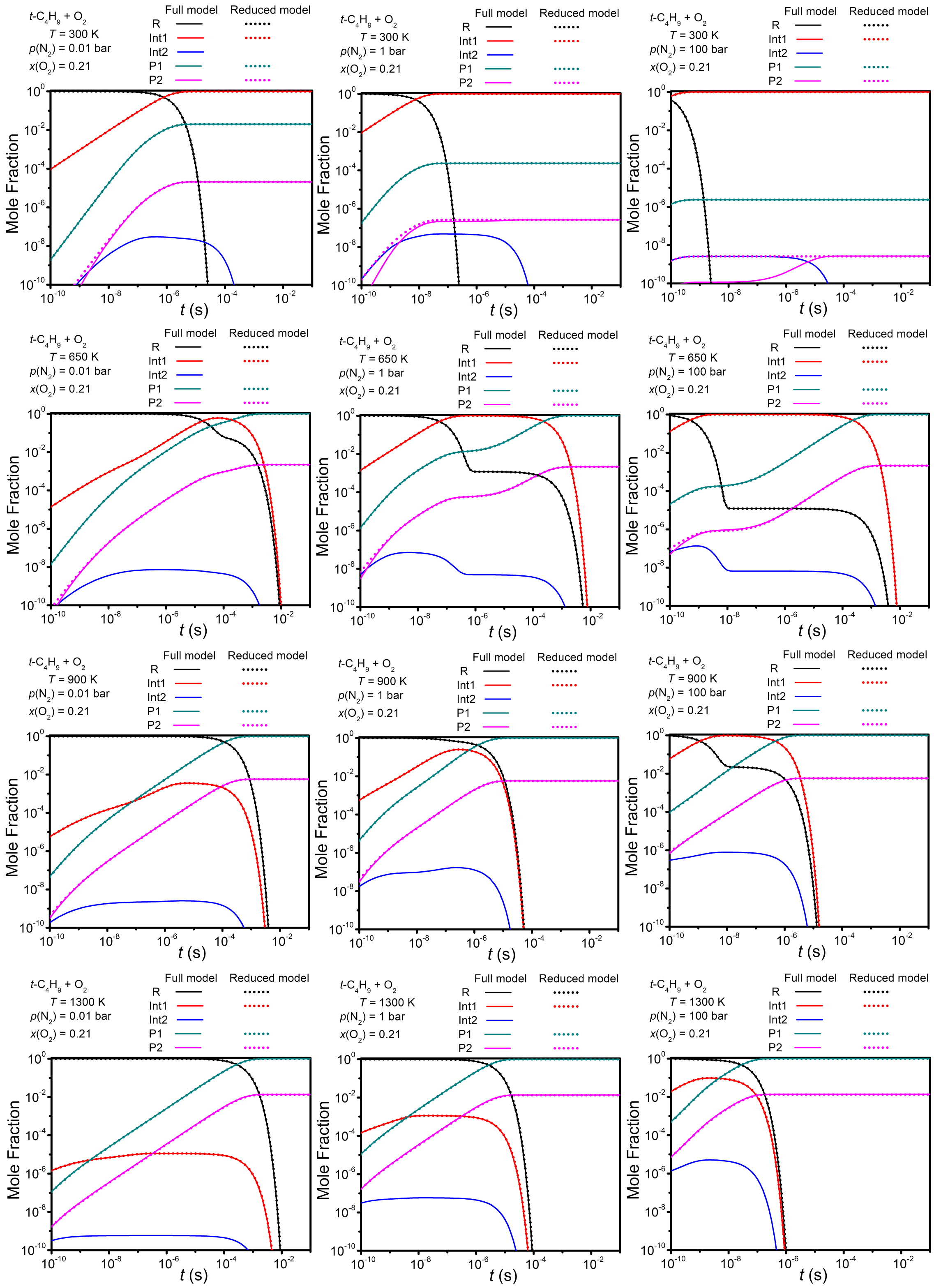

Figure S3: Concentration-time profiles simulated with the full (solid lines) and reduced (dotted lines) t-$C_4H_9^{\bullet}$ + O_2 models at different p_{N_2}, and T. Note that the profiles output by the two models nearly perfectly overlap.
